# How Does Routine Prostate-specific Membrane Antigen Positron Emission Tomography/Computed Tomography Modify the Current Management of Prostate Cancer? A Multidisciplinary View

**DOI:** 10.1016/j.euros.2025.03.007

**Published:** 2025-04-07

**Authors:** Daniela E. Oprea-Lager, Tessa van Elst, Shafak Aluwini, Els Dewulf, Henk van der Poel, Herman Stoevelaar, Chris H. Bangma, Aart Beeker, Steve Boudewijns, Tom Budiharto, Igle-Jan de Jong, Kim C. de Vries, Maarten L. Donswijk, Jurgen J. Fütterer, Paul Hamberg, Linda Heijmen, Robert J. Hoekstra, Thomas M.A. Kerkhofs, Jules Lavalaye, Daphne Luijendijk-de Bruin, Walter Noordzij, Irma M. Oving, Debbie G.J. Robbrecht, Eva E. Schaake, Addy C.M. van de Luijtgaarden, Roderick C.N. van den Bergh, Franchette van den Berkmortel, Tom van der Hulle, Johannes C.K. van der Mijn, Joyce M. van Dodewaard-de Jong, Michel van Kruchten, Pim J. van Leeuwen, Evert van Limbergen, R.Jeroen A. van Moorselaar, Inge M. van Oort, Joep G.H. van Roermund, Robert J. van Soest, Theo Veninga, André N. Vis, Jens Voortman, Peter-Paul M. Willemse, Derya Yakar, Niven Mehra

**Affiliations:** a Department of Radiology & Nuclear Medicine, Amsterdam University Medical Center, VU University, Amsterdam, The Netherlands; b Department of Medical Imaging, Radboud University Medical Centre, Nijmegen, The Netherlands; c Department of Urology, Canisius Wilhelmina Hospital, Nijmegen, The Netherlands; d Department of Medical Oncology, Radboud University Medical Centre, Nijmegen, The Netherlands; e Department of Radiation Oncology, University Medical Center Groningen, Groningen, The Netherlands; f Centre for Decision Analysis & Support, Ismar Healthcare NV, Lier, Belgium; g Department of Urology, Netherlands Cancer Institute-Antoni van Leeuwenhoek, Amsterdam, The Netherlands; h Department of Urology, Amsterdam University Medical Center, VU University, Amsterdam, The Netherlands; i Department of Urology, Erasmus MC, Rotterdam, The Netherlands; j Department of Internal Medicine, Spaarne Gasthuis, Hoofddorp, The Netherlands; k Department of Medical Oncology, Bravis Hospital Roosendaal, Roosendaal, The Netherlands; l Department of Radiotherapy, Catharina Hospital, Eindhoven, The Netherlands; m Department of Urology, University Medical Center Groningen, Groningen, The Netherlands; n Department of Radiotherapy, Erasmus MC, Rotterdam, The Netherlands; o Department of Nuclear Medicine, Netherlands Cancer Institute-Antoni van Leeuwenhoek, Amsterdam, The Netherlands; p Department of Medical Oncology, Franciscus Gasthuis & Vlietland, Rotterdam & Schiedam, The Netherlands; q Department of Radiology, Leiden University Medical Center, Leiden, The Netherlands; r Department of Urology, Catharina Hospital, Eindhoven, The Netherlands; s Department of Internal Medicine, Division of Medical Oncology, GROW School for Oncology and Developmental Biology, Maastricht University Medical Center+, Maastricht, The Netherlands; t Department of Nuclear Medicine, Sint Antonius Hospital, Utrecht & Nieuwegein, The Netherlands; u Department of Urology, Martini Hospital, Groningen, The Netherlands; v Department of Nuclear Medicine & Molecular Imaging, Medical Imaging Center Groningen, University of Groningen, University Medical Center Groningen, Groningen, The Netherlands; w Department of Internal Medicine, Ziekenhuis Groep Twente, Almelo, The Netherlands; x Department of Medical Oncology, Erasmus MC, Rotterdam, The Netherlands; y Department of Radiotherapy, Netherlands Cancer Institute-Antoni van Leeuwenhoek, Amsterdam, The Netherlands; z Department of Medical Oncology, Reinier Haga Prostate Cancer Centre, Delft, The Netherlands; aa Department of Internal Medicine-Medical Oncology, Zuyderland MC, Sittard, The Netherlands; bb Department of Medical Oncology, Leiden University Medical Center, Leiden, The Netherlands; cc Department of Medical Oncology, Netherlands Cancer Institute-Antoni van Leeuwenhoek, Amsterdam, The Netherlands; dd Department of Internal Medicine, Meander Medical Center, Amersfoort, The Netherlands; ee Department of Medical Oncology, University Medical Center Groningen, Groningen, The Netherlands; ff Department of Radiation Oncology (Maastro), GROW School for Oncology and Reproduction, Maastricht University Medical Centre+, Maastricht, The Netherlands; gg Department of Urology, Radboud University Medical Centre, Nijmegen, The Netherlands; hh Department of Urology, Maastricht University Medical Center+, Maastricht, The Netherlands; ii Department of Urology, Franciscus Gasthuis & Vlietland, Rotterdam & Schiedam, The Netherlands; jj Department of Radiotherapy, Institute Verbeeten, Tilburg, The Netherlands; kk Department of Medical Oncology, Amsterdam University Medical Center, VU University, Amsterdam, The Netherlands; ll Department of Urology, Cancer Center, University Medical Center Utrecht, Utrecht, The Netherlands; mm Department of Radiology, Medical Imaging Center Groningen, University of Groningen, University Medical Center Groningen, Groningen, The Netherlands; nn Department of Radiology, Netherlands Cancer Institute-Antoni van Leeuwenhoek, Amsterdam, The Netherlands

**Keywords:** Prostate cancer, Metastatic hormone-sensitive prostate cancer, Prostate-specific membrane antigen-positron emission tomography/computed tomography, Molecular hybrid imaging, Systemic therapy

## Abstract

**Background and objective:**

Prostate-specific membrane antigen positron emission tomography/computed tomography (PSMA-PET/CT) and new treatment modalities have expanded the possibilities for diagnosing and managing metastatic prostate cancer, but have also raised questions about their implementation in daily clinical practice. We sought consensus on definitions, preferred imaging modality for staging, and treatment selection in the era of next-generation imaging.

**Methods:**

A modified Delphi method involved two voting rounds and a face-to-face multidisciplinary meeting with 40 Dutch prostate cancer (PCa) experts. Consensus was reached if ≥75% of the panellists chose the same option. Appropriateness was assessed by the RAND Corporation/University of California Los Angeles appropriateness method.

**Key findings and limitations:**

There was consensus on performing metastatic screening with PSMA-PET/CT for unfavourable intermediate- or high-risk PCa. PSMA-PET/CT findings were considered feasible for determining treatment in synchronous metastatic hormone-sensitive prostate cancer, but there was no agreement on the validity of the CHAARTED criteria for interpreting the PSMA-PET/CT findings. If the PSMA-PET/CT findings led to upstaging after conventional imaging, 76% of panellists would opt for treatment intensification. In case of downstaging, 71% would choose for deintensification. Panellists would generally treat patients based on metastatic disease volume as per the CHAARTED criteria, except for bulky low-volume disease (LVD) and LVD with multiple (more than ten) bone metastases, all within the axial skeleton. This would be classified as LVD but treated as high-volume disease. Limitations are that the statements are largely consensus based and originate from a national (Dutch) perspective.

**Conclusions and clinical implications:**

PSMA-PET/CT was considered the preferred modality for initial PCa staging, which is nowadays the standard of care in The Netherlands. The majority of panellists would incorporate PSMA-PET/CT findings for treatment planning, including intensification and deintensification, but the criteria for interpreting metastatic disease volume on PSMA-PET/CT are still uncertain.

**Patient summary:**

A group of Dutch medical specialists discussed on how to diagnose metastatic hormone-sensitive prostate cancer and choose the most appropriate treatment for patients with this condition. It was concluded that imaging based on Prostate-specific membrane antigen (PSMA) positron emission tomography (PET) helps determine appropriate treatment options, with most experts supporting treatment adjustments based on PSMA-PET/computed tomography (CT) results. However, there is still some uncertainty about the criteria for interpreting the extent of metastatic disease with PSMA-PET/CT.

## Take Home Message

A multidisciplinary expert panel favoured prostate-specific membrane antigen positron emission tomography/computed tomography for primary staging of unfavourable intermediate- and high-risk prostate cancer and for selecting treatments in metastatic hormone-sensitive prostate cancer. The current disease volume classification needs to be adapted to the outcomes of this next-generation imaging modality.

## 1 Introduction

Over the past few years, the treatment landscape of metastatic hormone-sensitive prostate cancer (mHSPC) changed considerably. Upfront treatment intensification with prostate radiotherapy, docetaxel, and/or androgen receptor pathway inhibitors (ARPIs) has led to improved overall survival (OS), supplanting androgen deprivation therapy (ADT) monotherapy as the standard of care. Guidance for clinical decision-making is offered by (inter)national guidelines, but practice variation suggests that there is much room for interpretation [1]. Another factor contributing to practice variation is the increased use of next-generation imaging (NGI) in daily clinical practice. However, all level 1 evidence for treatment decision-making is based on staging with conventional imaging (CI), and it remains unclear whether the results of landmark trials can be extrapolated to patients diagnosed with NGI.

Prostate-specific membrane antigen (PSMA) positron emission tomography (PET)/computed tomography (CT) has superior accuracy to CI (bone and CT scan) to stage men with high-risk prostate cancer (PCa) [2]. However, the role of PSMA-PET/CT is less established in metastatic PCa, due to its anticipated stage migration and lack of implementation studies of PSMA-PET/CT alongside CI [3], [4]. Owing to its greater accuracy, PSMA-PET/CT has been adopted in Dutch clinical practice as the standard for PCa staging. Nevertheless, the starting points for treatment decisions are still formed by the CHAARTED criteria for metastatic spread and disease volume that are based on CI [1], [5]. As PSMA-PET/CT often leads to upgrading of the disease volume, this raises important questions for therapeutic consequences.

A Dutch panel study was conducted to explore the consensus on potential implications of NGI for therapeutic decisions in synchronous mHSPC.

## 2 Patients and methods

### 2.1 Study design

A multidisciplinary scientific committee and an advising methodologist set up the consensus meeting. The approach combined elements from the Delphi method, Nominal Group Technique, and consensus development techniques [6].

### 2.2 Panel composition

The panel consisted of 14 medical oncologists, 13 urologists, six radiation oncologists, five nuclear medicine physicians, and two radiologists. The selection criteria included clinical and scientific expertise in the field of PCa, geographic spread, distribution between academic and peripheral hospitals, availability to participate in both voting rounds, and face-to-face consensus meeting.

### 2.3 Explorative survey

Based on a literature search, data from a Dutch explorative mixed-method study (NOTES [1]), and clinical expertise of the Scientific Committee members, an explorative survey was prepared, including statements and questions on imaging, interpretation of imaging results, and their meaning for treatment decisions. Questions on treatment choice were related to seven clinical scenarios with the same basic characteristics (T3a, prostate-specific antigen 50 ng/ml, and International Society of Urological Pathology [ISUP] 4), but with varying outcomes of imaging (type, number, and location of metastases). Panellists were asked to complete the survey, and provide detailed feedback and suggestions for improvement.

### 2.4 Consensus meeting

The outcomes of the explorative survey were discussed during a face-to-face meeting that took place in Den Dolder (The Netherlands) on February 2, 2024. The discussion was preceded by state-of-the-art lectures on the use of NGI modalities to diagnose mHSPC and the therapeutic landscape of mHSPC. This ensured that all panellists could start from the same level of scientific evidence. The scientific committee members and an advising nonvoting methodologist led the discussion. During the discussion, proposals for adaptations were made. The refined clinical scenarios and statements were sent out in a second survey, 1 wk after the face-to-face meeting. This survey contained 107 statements/questions, 25 with a multiple-choice format and 82 with rating the appropriateness of options on a 9-point scale (1–3: inappropriate, 4–6: uncertain, and 7–9: appropriate). Eight clinical case scenarios were included, one on interpretation of imaging results and seven on treatment decisions. All items included the option “cannot judge” in case the expert lacked experience for a specific question or felt unable to vote for any other reason.

### 2.5 Statistical analysis

Appropriateness was calculated using the mathematical rules typically applied in studies on the RAND Corporation/University of California Los Angeles appropriateness method [7]. An option was considered appropriate if the median panel score was between 7 and 9, and inappropriate if the median was between 1 and 3, in the absence of disagreement. Disagreement was defined as the situation in which at least one-third of the panellists scored in each of the sections 1–3 and 7–9. All other outcomes were deemed “equivocal/uncertain”. For other multiple-choice questions, strong agreement (consensus) was defined as the situation in which ≥75% of the panellists chose the same option. If the option “cannot judge” was chosen, the answer was excluded from the agreement calculations.

## 3 Results

Consensus was reached in 76% of the items. For the appropriateness aspects, disagreement was seen in 4% of the ratings.

### 3.1 Diagnosis—imaging modalities

There was consensus that for primary staging of favourable intermediate-risk PCa, no metastatic screening should be performed (85%), and that metastatic screening using PSMA-PET-based imaging should be performed for patients with unfavourable intermediate-risk (97%) or high-risk (100%) PCa (Table 1). For patients with intermediate-risk PCa, use of PSMA-PET/CT was considered uncertain (disagreement) if ISUP grade 2 was combined with cribriform pattern in prostate tissue (Table 2). Both PSMA-PET/CT and PSMA-PET/magnetic resonance imaging (MRI) were considered appropriate options for routine staging of patients with high-risk PCa (Table 2). The main reasons mentioned to deviate from PSMA-PET/CT included the absence of treatment consequences (85%), metastases identified previously by another imaging modality (68%), and limited life expectancy of the patient (65%; Supplementary material). Utilisation of the PROMISE criteria [8] was considered appropriate to systematically record PSMA-PET/CT findings (Table 2). For high-risk PCa patients having bone lesions on PSMA-PET/CT, additional targeted MRI was recommended in case of a doubt regarding metastases (82%), and if there is a suspicion of benign findings (eg, bone fracture [68%] or spinal cord compression [100%]; Supplementary material).

**Table 1:** Panel results on the appropriateness of different imaging modalities for primary staging (detection of metastases) in PCa (*N* = 40 panellists)^a^

Question	Panellists (%)^a^, ^b^	Cannot judge (%)
Which imaging modality do you consider most appropriate in the current, average Dutch practice for primary staging (detection of metastases) for favourable-risk intermediate PCa?		18
Bone and CT scan	3	
PSMA-PET/CT or PSMA-PET/MRI	12	
Whole-body MRI	0	
No imaging for metastatic screening	**85**	
Which imaging modality do you consider most appropriate in the current, average Dutch practice for primary staging (detection of metastases) for unfavourable-risk intermediate PCa?		5
Bone and CT scan	0	
PSMA-PET/CT or PSMA-PET/MRI	**97**	
Whole-body MRI	0	
No imaging for metastatic screening	3	
Which imaging modality do you consider most appropriate in the current, average Dutch practice for primary staging (detection of metastases) for high-risk PCa?		3
Bone and CT scan	0	
PSMA-PET/CT or PSMA-PET/MRI	**100**	
Whole-body MRI	0	
No imaging for metastatic screening	0	

CT = computed tomography; MRI = magnetic resonance imaging; PCa = prostate cancer; PET = positron emission tomography; PSMA = prostate-specific membrane antigen.

a The bold values represent statements for which ≥75% of the panellists chose the same option (consensus).

b Percentage of valid answers, that is, after the exclusion of the “cannot judge” category.

**Table 2:** Panel appropriateness outcomes on the diagnosis and the interpretation of diagnzstic results (*N* = 40 panellists)^a^

CT = computed tomography; D = disagreement: at least one-third of the scores in each of the sections 1–3 and 7–9; ISUP = International Society of Urological Pathology; mHSPC = metastatic hormone-sensitive prostate cancer; MRI = magnetic resonance imaging; PCa = prostate cancer; PET = positron emission tomography; PSA = prostate-specific antigen; PSMA = prostate-specific membrane antigen.

a Appropriateness: green—appropriate (median score 7–9, no disagreement), red—inappropriate (median score 1–3, no disagreement), and yellow—uncertain (median score 4–6 and/or disagreement).

### 3.2 Interpretation of imaging results

PSMA-PET/CT was considered appropriate to determine treatment preference in patients with synchronous mHSPC (Table 2). There was no agreement that the CHAARTED definition is also valid for the interpretation of PSMA-PET/CT findings (Table 2). The following parameters were considered important to define disease extent based on PSMA-PET/CT: number of lesions (85%), presence of oligo- or polymetastases (53%), total tumour volume (50%), and location of metastatic lesions (43%; Supplementary material). Opinions on the meaning of bone uptake on PSMA-PET, without anatomical substrate on CT, for defining disease volume were dispersed (Table 2, Table 3). If PSMA-PET/CT results would lead to upstaging after CI, 76% of panellists would opt for treatment intensification. In case of downstaging, 71% would choose for deintensification (Supplementary material).

**Table 3:** Clinical scenario on the interpretation of PSMA-PET/CT results without anatomical substrate on CT (*N* = 40 panellists)

Six bone lesions are seen on PSMA-PET/CT of a patient: 1. Five bone lesions are localised within the axial skeleton, of which two have an anatomical substrate on CT scan 2. One bone lesion is localised outside the axial skeleton with anatomical substrate on CT scan		

Question	Panellists (%)^a^	Cannot judge (%)

How would you treat this patient?		10
1. As having low-volume disease	56	
2. As having high-volume disease	44	

CT = computed tomography; PET = positron emission tomography; PSMA = prostate-specific membrane antigen.

a Percentage of valid answers, that is, after the exclusion of the “cannot judge” category.

### 3.3 Treatment choice

**Fig. 1:**
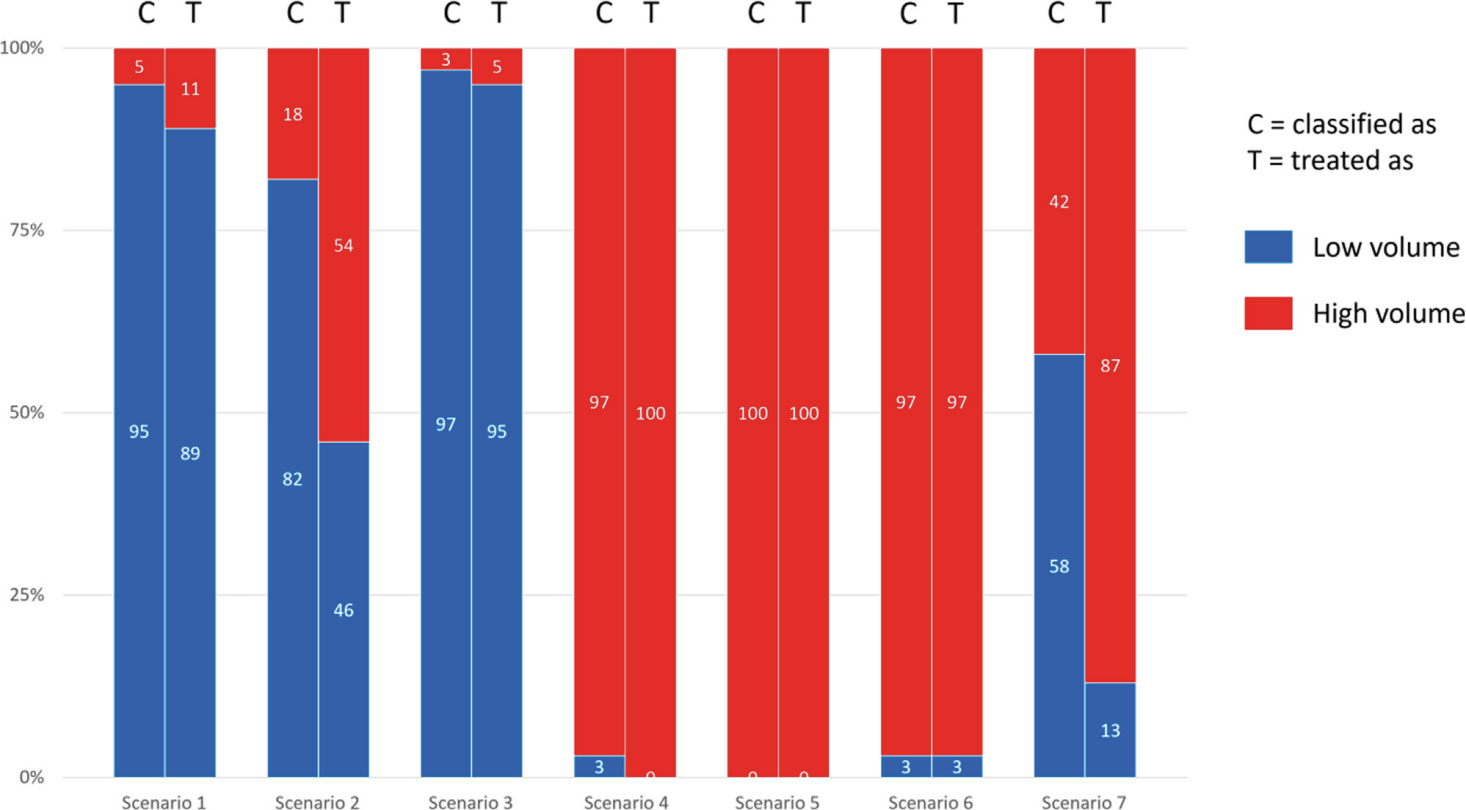
Opinions of the panellists on the classification of disease volume (low vs high) and treatment choice according to disease volume (low vs high) for different scenarios. Figures are exclusive of the option “cannot judge” (between 2.5% and 7.5% per question). The following findings were seen on PSMA-PET/CT: scenario 1: multiple pathologically enlarged lymph nodes (more than ten, short axis diameter max 2 cm, all above the iliac and aorta bifurcation); scenario 2: multiple significantly enlarged bulky lymph nodes (short axis 5–7 cm, also above iliac and aorta bifurcation); scenario 3: two bone metastases (L1, acetabulum) and two locoregional lymph nodes (parailiac, below the iliac bifurcation); scenario 4: two bone metastases (L1, acetabulum), two locoregional lymph nodes (parailiac, below the iliac bifurcation), and two lung metastases; scenario 5: two bone metastases (L1, acetabulum), two locoregional lymph nodes (parailiac, below the iliac bifurcation), and two liver metastases; scenario 6: six bone metastases (five spinal metastases L1–4 and one proximal left humerus) and several locoregional lymph nodes (all below iliac bifurcation); scenario 7: ten bone metastases (all within axial skeleton) and several locoregional lymph nodes (all below iliac bifurcation). For all scenarios, PSMA-avid lesions had an anatomical substrate on CT. CT = computed tomography; PET = positron emission tomography; PSMA = prostate-specific membrane antigen.

The panellists would mostly treat patients according to disease volume classification, except for scenarios 2 and 7 (Fig. 1). Most uncertainty regarding treatment preference was found for the scenario with bulky M1a disease (Table 4, scenario 2). For all scenarios, ADT monotherapy was deemed inappropriate, while doublet therapy of ADT + ARPI was considered an appropriate option. Both the combination of ADT + docetaxel and the triplet therapy (ADT + docetaxel + ARPI) were considered appropriate for high-volume mHSPC, but never for low-volume disease (LVD). To choose between different ARPIs, the following factors were considered to be most important: comorbidities, comedication, Eastern Cooperative Oncology Group performance status, life expectancy, and frailty in case of elderly patients; patient age, pain/discomfort of local disease, and/or metastases were considered less important factors (Supplementary material). The different ARPIs were considered to be equally appropriate, regardless of disease volume (low vs high, CHAARTED) and risk (low vs high, LATITUDE), provided that it is in accordance with the applicable registration (Supplementary material).

**Table 4:** Panel appropriateness outcomes for treatment options of different patient scenarios diagnosed with synchronous mHSPC^a^, ^b^




ADT = androgen deprivation therapy; ARPI = androgen receptor pathway inhibitor; CT = computed tomography; D = disagreement: at least one-third of the scores in each of the sections 1–3 and 7–9; DRE = digital rectal examination; ECOG PS = Eastern Cooperative Oncology Group performance status; ISUP = International Society of Urological Pathology; mHSPC = metastatic hormone-sensitive prostate cancer; mpMRI = multiparametric magnetic resonance imaging; PET = positron emission tomography; PSMA = prostate-specific membrane antigen; PSA = prostate-specific antigen; RTx = radiotherapy to the prostate.

a For all scenarios, all PSMA-avid lesions had an anatomical substrate on CT (*N* = 40 panellists).

b Appropriateness: green—appropriate (median score 7–9, no disagreement), red—inappropriate (median score 1–3, no disagreement), and yellow—uncertain (median score 4–6 and/or disagreement).

## 4 Discussion

### 4.1 Diagnosis—imaging modalities

There was consensus that metastatic screening with PSMA-PET/CT should be performed in men with unfavourable intermediate- or high-risk PCa. Likewise, experts participating in a consensus meeting of the European Association of Nuclear Medicine (EANM) Focus 5 also agreed that PSMA-PET/CT should replace CI in patients with high-risk PCa undergoing initial staging [9].

The European Association of Urology (EAU) PCa guidelines strongly recommend metastatic screening using PSMA-PET/CT, if available, and at least CT and bone scan for high-risk disease, while this is only a weak recommendation for men with intermediate-risk and ISUP grade 3 disease [10]. The guidelines emphasise that when NGI is used for staging of PCa to increase sensitivity, one should be aware of the lack of outcome data of subsequent treatment changes [10].

NGI has shown higher sensitivity and specificity than CI for initial staging of men with localised PCa [2], [11]. The increased use of NGI may therefore increase the prevalence of synchronous mHSPC. In the ProPSMA trial, including high-risk PCa patients, PSMA-PET/CT was 27% more accurate than CI for staging, mainly due to increased sensitivity, and it led to a change in management in 28% of patients [2].

There was disagreement among the panellists on whether PSMA-PET/CT should be performed in case of intermediate-risk PCa with ISUP grade 2 and cribriform growth in the prostate, as cribriform pattern is associated with unfavourable oncological outcomes. Recent data have demonstrated invasive cribriform growth to be a significant predictor of biochemical recurrence-free survival [12]. However, a Dutch retrospective study found that cribriform pattern in biopsies was not an independent risk factor for metastatic disease on ^68^Ga-PSMA-PET/CT [13].

### 4.2 Interpretation of imaging results

Despite the higher sensitivity of PSMA-PET/CT versus CI to detect metastatic disease, PSMA-PET/CT might also indicate false-positive findings [14]. When comparing different PSMA radiotracers, it was shown that ^18^F-PSMA-1007 results in a significantly higher rate of equivocal and false-positive findings due to nonspecific bone uptake [15], [16], [17]. This requires a learning curve and higher degree of reader experience for proper interpretation. However, a Dutch study showed that the interobserver agreement was moderate when using the ^18^F-PSMA-1007 tracer and not impaired by nonspecific bone uptake [18]. False-positive findings can lead to incorrect (up)staging of the patient, with a negative impact on the outcomes and a need for additional diagnostic workup, such as extra imaging or a confirmation biopsy. When having doubts whether bone lesions on PSMA-PET/CT represent bone metastases, the majority of panellists would opt for further evaluation through targeted MRI. This is in line with the recently published 2024 Advanced Prostate Cancer Consensus Conference (APCCC) recommendation to perform additional imaging or a biopsy in such a case [19]. The opinions on the meaning of bone lesions without CT correlate to determine the disease volume differed substantially (44% in favour and 56% against). In a small, retrospective, PSMA-PET/CT-guided biopsy study, none of the nonspecific bone uptake lesions had a CT correlate and only one lesion was a confirmed bone metastasis [16]. The use of the standardised evaluation criteria for whole-body imaging, such as PROMISE, would offer a harmonised framework for PSMA-PET interpretation [8].

The panellists considered the use of PSMA-PET/CT appropriate to select treatment in patients with synchronous mHSPC. However, the use of the CHAARTED criteria to define high-volume disease (HVD) versus LVD based on PSMA-PET/CT results was considered uncertain. There is an unmet need to define disease extent on PSMA-PET/CT that is useful in daily clinical practice. When asked about the parameters to be included in this definition, the panel favoured the number of lesions over the presence of oligo- or polymetastases, total tumour volume, and location of metastatic lesions. There were no pronounced preferences for specific combinations of these four variables (Supplementary material). Although the panel considered the number of lesions to be most important in determining the disease extent and treatment plan, total tumour volume is the most accurate parameter, as ten small metastatic lesions on PSMA-PET/CT might correspond to three larger lesions on CI, still resulting in LVD. A retrospective study showed that volume quantification with PSMA-PET delivered comparable discrimination between LVD and HVD, with additional subgroups for unifocal, oligometastatic, and disseminated disease for guiding therapy [20]. Metastatic disease volume, by adding all PSMA-positive lesions, could define a linear scale for disease activity and could omit the need for classification in LVD and HVD. Quantification of total molecular volume has been shown to be efficient and robust, and seems to be prognostic for OS [21], [22]. In addition, tumour volume measurements are part of the PROMISE criteria [8].

Owing to the higher accuracy of PSMA-PET/CT versus CI, NGI may lead to both upstaging and downstaging of the disease [23]. Of the panellists, 76% would intensify treatment if upstaging occurs (55% in most cases and 21% in specific occasions). Specific occasions include the presence of visceral metastases on PSMA-PET/CT, presence of metastases with evident anatomical substrate on CT, a logical metastatic pattern on imaging, and absence of nonspecific bone uptake on PSMA-PET (Supplementary material). In case of downstaging, 71% of the panellists would opt for treatment deintensification. An Italian study, in which patients underwent both CI and PSMA-PET/CT (though managed according to CI staging), showed that PSMA-PET/CT outcomes better predicted biochemical recurrence, suggesting these to be superior in choosing between local and systemic treatment [23].

### 4.3 Treatment choice

The panellists’ opinions on the *classification* of disease volume (low vs high) and related *treatment choice* (as having LVD or HVD) were discordant for two out of seven clinical scenarios (Fig. 1). For patients with bulky lymphadenopathy (scenario 2), most panellists *classified* this scenario as LVD, but less than half of them would have *treated* these accordingly. An even more pronounced effect was seen for patients with multiple bone metastases located solely in the axial skeleton (scenario 7). Whereas less than half of the panellists *classified* this scenario as LVD, the majority would have *treated* these patients as having HVD. These discrepancies suggest that the current classification of disease volume may not be fully adequate. The CHAARTED definition of LVD represents a heterogeneous group of patients, ranging from patients having M1a disease to those having multiple bone metastases within the axial skeleton [5]. This heterogeneity leads to differences in treatment patterns across patients having LVD based on the CHAARTED definition. In addition, the results of the NOTES study showed that most practice variation is seen for patients with LVD [1]. Contributing factors included the heterogeneous aspect of LVD, ambiguous definitions, differences in the interpretation of metastatic volume on PSMA-PET/CT, and guideline gaps [1]. These outcomes reflect the need to adopt harmonised PSMA-PET/CT reporting criteria in daily clinical practice and the unmet demand to assess disease volume when using NGI modalities. For patients with HVD (scenarios 4–6), in addition to ADT + ARPI ± docetaxel, ADT + docetaxel was still considered a suitable option, aligning with the updated Dutch guidelines on PCa management published in April 2024 [24]. However, the EAU PCa guidelines no longer recommend docetaxel in doublet therapy, but only in triplet therapy [25]. The APCCC favoured triplet therapy much more for (at least) selected patients with high-burden than those with low-burden disease [19]. Triplet therapy, studied in PEACE-1 (ADT + docetaxel + abiraterone) and ARASENS (ADT + docetaxel + darolutamide), showed significant survival benefits versus ADT + docetaxel [26], [27]. In addition, patient-reported quality of life was superior in those who received ADT + ARPI than in those who received ADT + docetaxel in the STAMPEDE trial [28]. Therefore, the use of ADT + docetaxel should not be considered if ADT + ARPI is available as an alternative. However, the benefits of ADT + ARPI over ADT + docetaxel should be balanced against the duration of treatment, which might be associated with increased financial toxicity, increased cardiovascular morbidity and mortality, and/or other chronic physical toxicities [29].

### 4.4 National versus international perspective

The importance of NGI for staging mHSPC is also reflected in the results from other (international) consensus studies such as the EANM Focus 5 [9] and the 2024 APCCC [19], and many of our recommendations are similar. In our view, the added value of our national consensus firstly lies in the specific local situation where PSMA-PET/CT is used routinely for staging PCa, which was taken as a starting point for our panel. This is different from other international consensus studies, where panel members are mostly representing different continents and countries, with different levels of adoption of NGI. Our recommendations therefore have a more homogeneous starting point, but these also show the uncertainty about treatment implications. Actually, it shows what happens when practice is ahead of evidence. Using an approach with consecutive scenarios (from a low to high burden), we have tried to explore the consensus on specific treatment decisions in light of the routine use of NGI for (re)staging of mHSPC.

### 4.5 Limitations

This national panel study focused on an area where local practice with next NGI is ahead of evidence regarding implications for treatment choice. The outcomes are therefore largely consensus based. Furthermore, this panel study lacked a comprehensive systematic literature review. Finally, the relatively small number of participants from specific disciplines may have skewed the results.

## 5 Conclusions

PSMA-PET/CT is considered appropriate for primary staging of unfavourable intermediate- and high-risk PCa, and for selecting treatments in patients with synchronous mHSPC. The majority of panellists would (de)intensify treatment based on the PSMA-PET/CT findings, though the criteria for interpreting metastatic disease volume on PSMA-PET/CT remain uncertain.

  ***Author contributions:*** Daniela E. Oprea-Lager had full access to all the data in the study and takes responsibility for the integrity of the data and the accuracy of the data analysis.

  *Study concept and design*: Oprea-Lager, Aluwini, Dewulf, van der Poel, Stoevelaar, Yakar, Mehra.

*Acquisition of data*: Oprea-Lager, van Elst, Aluwini, Dewulf, van der Poel, Stoevelaar, Yakar, Mehra.

*Analysis and interpretation of data*: Oprea-Lager, van Elst, Aluwini, Dewulf, van der Poel, Stoevelaar, Yakar, Mehra.

*Drafting of the manuscript*: Dewulf, Stoevelaar, Oprea-Lager, van Elst, Mehra.

*Critical revision of the manuscript for important intellectual content*: Aluwini, van der Poel, Yakar.

*Statistical analysis*: Stoevelaar, Dewulf.

*Obtaining funding*: Oprea-Lager, Aluwini, Mehra, van der Poel, Yakar.

*Administrative, technical, or material support*: Dewulf, Stoevelaar.

*Supervision*: Stoevelaar.

*Other*: Consensus ratings, review statements, review manuscript: Bangma, Beeker, Boudewijns, Budiharto, de Jong, de Vries, Donswijk, Fütterer, Hamberg, Heijmen, Hoekstra, Kerkhofs, Lavalaye, Luijendijk-de Bruin, Noordzij, Oving, Robbrecht, Schaake, van de Luijtgaarden, van den Bergh, van den Berkmortel, van der Hulle, van der Mijn, van Dodewaard-de Jong, van Kruchten, van Leeuwen, van Limbergen, van Moorselaar, van Oort, van Roermund, van Soest, Veninga, Vis, Voortman, Willemse.

  ***Financial disclosures:*** Daniela E. Oprea-Lager certifies that all conflicts of interest, including specific financial interests and relationships and affiliations relevant to the subject matter or materials discussed in the manuscript (eg, employment/affiliation, grants or funding, consultancies, honoraria, stock ownership or options, expert testimony, royalties, or patents filed, received, or pending), are the following: Daniela E. Oprea-Lager has received institutional consultancy fee from Astellas during the conduct of the study, and two unrestricted grants from Janssen in 2020 and 2022 outside the submitted work; institutional research support from Curium. Tessa van Elst is PhD trajectory funded by Janssen-Cilag BV. Shafak Aluwini has received a consultancy fee from Astellas. Els Dewulf is an employee of Ismar Healthcare NV. Henk van der Poel has received a consultancy fee from Astellas. Herman Stoevelaar is a partner of Ismar Healthcare NV. Igle-Jan de Jong is an educational consultant for Bayer and AstraZeneca. Paul Hamberg has received consulting or advisory fees from Astellas, MSD, Pfizer AstraZeneca, BMS, Ipsen, and Janssen. Addy C.M. van de Luijtgaarden have received travel expenses from Ipsen; consultancy for Janssen and Astellas. Roderick C.N. van den Bergh is a speaker honoraria from Amgen, Astellas, Ipsen, Janssen, and MSD; has received travel grants from Astellas; received research support from Astellas and Janssen; and participated in trials run by Janssen. Evert van Limbergen has received fees from Varian (Siemens Healthineers Company) and speaker fee from Johnson and Johnson (institutional). R. Jeroen A. van Moorselaar is a consultant or advisor for Astellas, AstraZeneca, Bayer, BMS, Curium, Janssen, and Pantarhei Bioscience. Inge M. van Oort has received fees from Astellas, Bayer, MSD/AstraZeneca, Novartis/AAA, and Pfizer. Robert J. van Soest has received honoraria from Astellas, Bayer, Johnson and Johnson, Sanofi Genzyme, MSD, and AstraZeneca. Derya Yakar has received a consultancy fee from Astellas; research support from Siemens Healthineers, Health Hollond, NWO, and Hanarth fund; honoraria from Astellas; consulting fees and travel grant from MDPI Life Journal; and speaker fees from Bayer. Niven Mehra has received consultancy fees from Astellas; research support from Astellas, Janssen, AstraZeneca/MSD, Pfizer, BMS, and Roche; and honoraria from AstraZeneca/MSD, Pfizer, BMS, Janssen, Astellas. The remaining authors have nothing to disclose.

  ***Funding/Support and role of the sponsor:*** The consensus study was supported by Astellas Pharma B.V., but this company had no influence on the content in any stage of the process.

  ***Acknowledgements:*** The authors are grateful to Margot Van Riel and Hanne Kraat from Ismar Healthcare NV for supporting the consensus study and meeting. Panel members (in alphabetic order by specialty): medical oncology: Aart Beeker (Spaarne Gasthuis), Steve Boudewijns (Bravis Hospital Roosendaal), Paul Hamberg (Franciscus Gasthuis & Vlietland), Thomas Kerkhofs (Maastricht University Medical Center+), Niven Mehra (Radboud University Medical Centre), Irma Oving (Ziekenhuis Groep Twente), Debbie Robbrecht (Erasmus MC), Addy van de Luijtgaarden (Reinier Haga Prostate Cancer Centre), Franchette van den Berkmortel (Zuyderland MC), Tom van der Hulle (Leiden University Medical Center), Koen van der Mijn (Netherlands Cancer Institute – Antoni van Leeuwenhoek), Joyce van Dodewaard-de Jong (Meander Medical Center), Michel van Kruchten (University Medical Center Groningen), Jens Voortman (Amsterdam University Medical Center); nuclear medicine: Maarten Donswijk (Netherlands Cancer Institute – Antoni Van Leeuwenhoek), Linda Heijmen (Leiden University Medical Center), Jules Lavalaye (Sint Antonius Hospital), Walter Noordzij (University Medical Center Groningen), Daniela Oprea-Lager (Amsterdam University Medical Center); radiology: Jurgen Fütterer (Radboud University Medical Centre), Derya Yakar (University Medical Center Groningen & Netherlands Cancer Institute – Antoni van Leeuwenhoek); radiation oncology: Shafak Aluwini (University Medical Center Groningen), Tom Budiharto (Catharina Hospital), Kim de Vries (Erasmus MC), Eva Schaake (Netherlands Cancer Institute – Antoni van Leeuwenhoek), Evert van Limbergen (Maastro/Maastricht University Medical Center+), Theo Veninga (Institute Verbeeken); urology: Chris Bangma (Erasmus MC), Igle-Jan de Jong (University Medical Center Groningen), Robert Hoekstra (Catharina Hospital), Daphne Luijendijk-de Bruin (Martini Hospital Groningen), Roderick van den Bergh (Erasmus MC), Henk van der Poel (Netherlands Cancer Institute – Antoni van Leeuwenhoek & Amsterdam University Medical Center), Pim van Leeuwen (Netherlands Cancer Institute – Antoni van Leeuwenhoek), Jeroen van Moorselaar (Amsterdam University Medical Center), Inge van Oort (Radboud University Medical Centre), Joep van Roermund (Maastricht University Medical Center+), Robert van Soest (Franciscus Gasthuis & Vlietland), André Vis (Amsterdam University Medical Center), and Peter-Paul Willemse (University Medical Center Utrecht). Presenters at the face-to-face consensus meeting: Niven Mehra, Daniela Oprea-Lager, Tessa van Elst, and Derya Yakar. Scientific Committee: Shafak Aluwini, Niven Mehra, Daniela Oprea-Lager, Henk van der Poel, and Derya Yakar.
