# Supplementary material for: How Does Routine Prostate-specific Membrane Antigen Positron Emission Tomography/Computed Tomography Modify the Current Management of Prostate Cancer? A Multidisciplinary View
Source: Eur Urol Open Sci. 2025 Apr 7;75:69–79. doi: 10.1016/j.euros.2025.03.007 (PMC12008539; doi:10.1016/j.euros.2025.03.007)
Supplement: Supplementary Data 1 [file mmc1.pdf]

SUPPLEMENTARY MATERIALS

Statements, questions and clinical scenarios

Part 1: diagnosis

Question 1a

Which imaging modality do you consider most appropriate in the current, average Dutch practice for primary staging (detection of metastases) for:
favourable intermediate-risk prostate cancer [1 answer option possible]

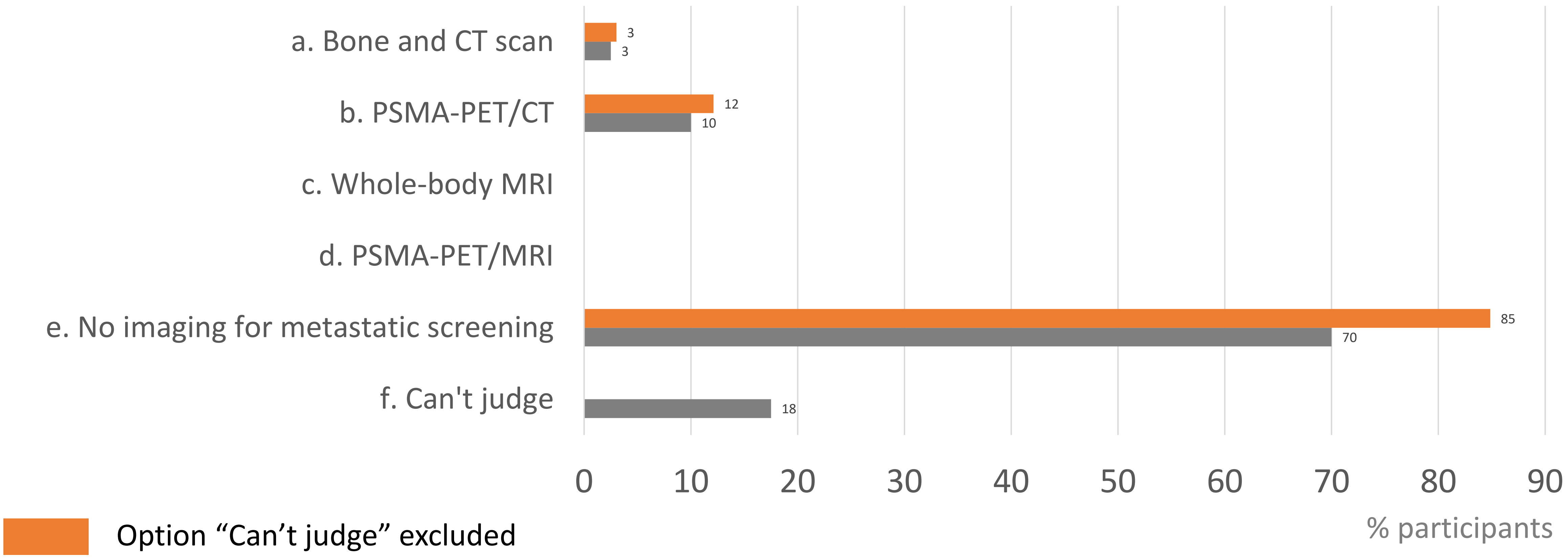

Question 1b

Which imaging modality do you consider most appropriate in the current, average Dutch practice for primary staging (detection of metastases) for:
unfavourable intermediate-risk prostate cancer [1 answer option possible]

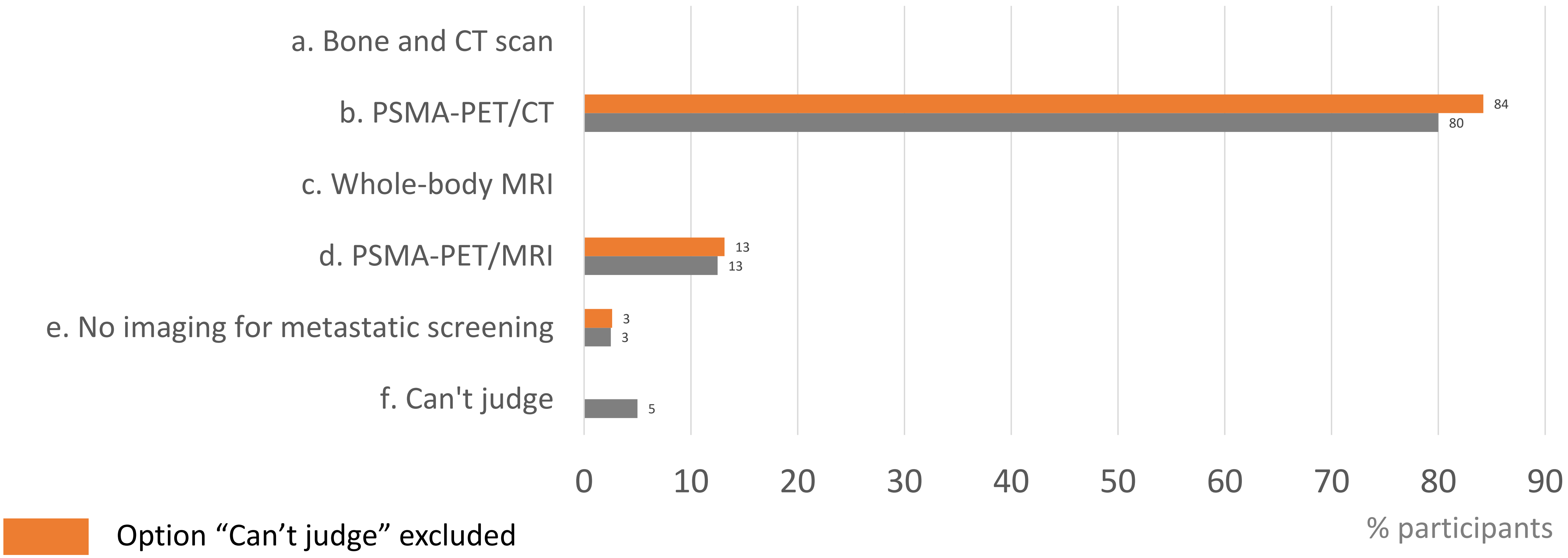

Consensus

Question1c

Which imaging modality do you consider most appropriate in the current, average Dutch practice for primary staging (detection of metastases) for:
high-risk prostate cancer [1 answer option possible]

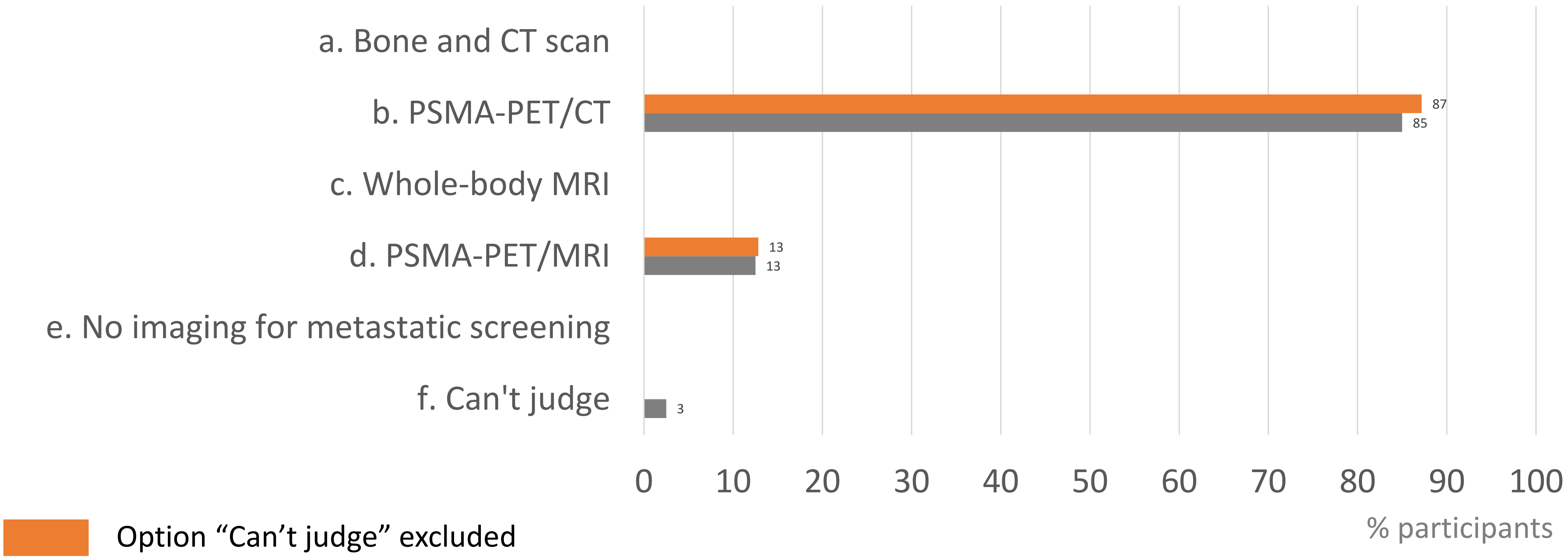

Question 2

How appropriate do you consider the use of PSMA-PET/CT for primary staging (detection of metastases) in patients with:

Options	Appropriateness[1] (median)	Can't judge[2] (%)
a. Intermediate-risk prostate cancer and ISUP grade 2	2.0	20
b. Intermediate-risk prostate cancer and ISUP grade 3	7.0	10
c. Intermediate-risk prostate cancer and Gleason score 3+4 (ISUP grade 2) and cribriform pattern in the prostate	5.0*	13

1 Score on a 9-point scale: 1-3 inappropriate (red), 4-6 uncertain or disagreement (yellow), 7-9 appropriate (green)
2 Total number of respondents=40
*Disagreement: at least one-third of the scores in each of the sections 1-3 and 7-9

Question 3

How appropriate do you consider the following imaging modalities for primary staging (detection of metastases) for patients with high-risk prostate cancer?

Options	Appropriateness[1] (median)	Can't judge[2] (%)
a. Bone and CT scan	5.0	5
b. PSMA-PET/CT	9.0	0
c. Whole-body MRI	5.5	25
d. PSMA-PET/MRI	8.0	28

[1] Score on a 9-point scale: 1-3 inappropriate (red), 4-6 uncertain or disagreement (yellow), 7-9 appropriate (green)
[2] Total number of respondents=40

Question 4

In which specific situations do you consider it necessary to deviate from PSMA-PET/CT as routine imaging technique? [checkbox, multiple options possible]

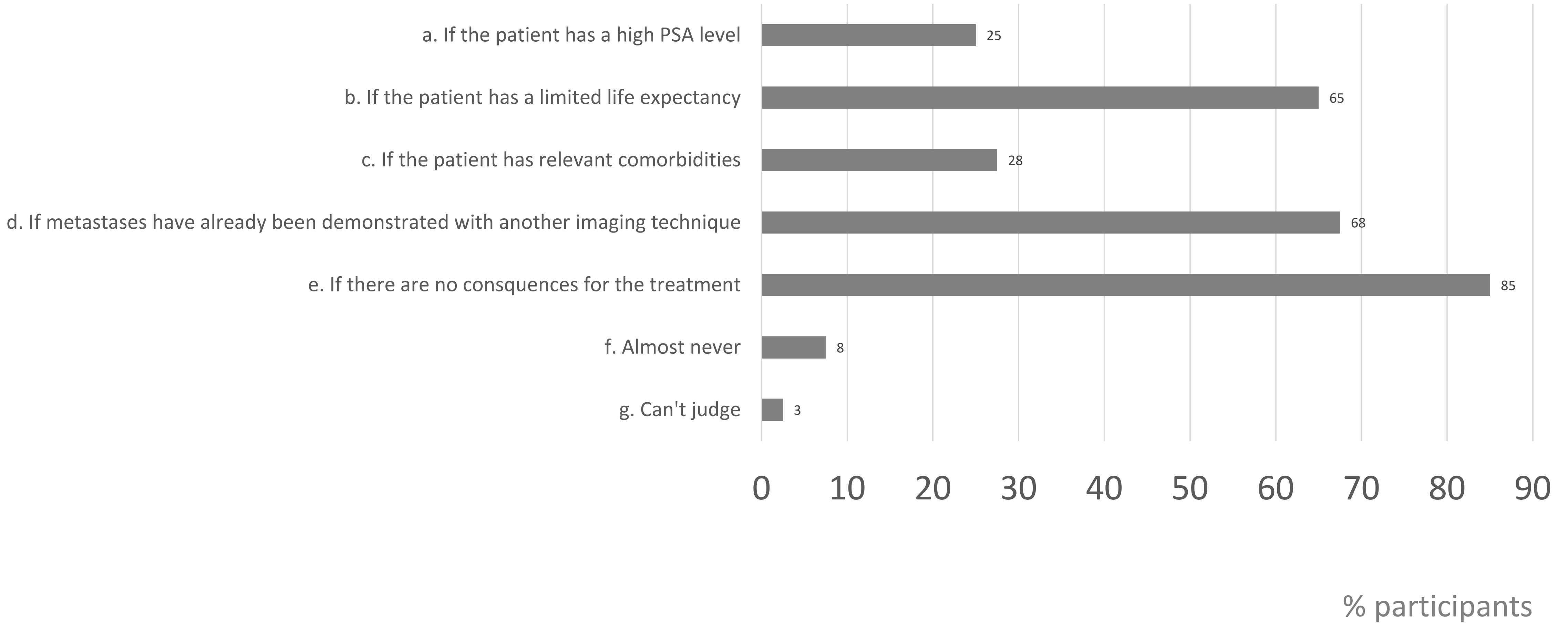

Question 4b

If you answered “If the patient has a high PSA level” (N=10), please specify: higher than…

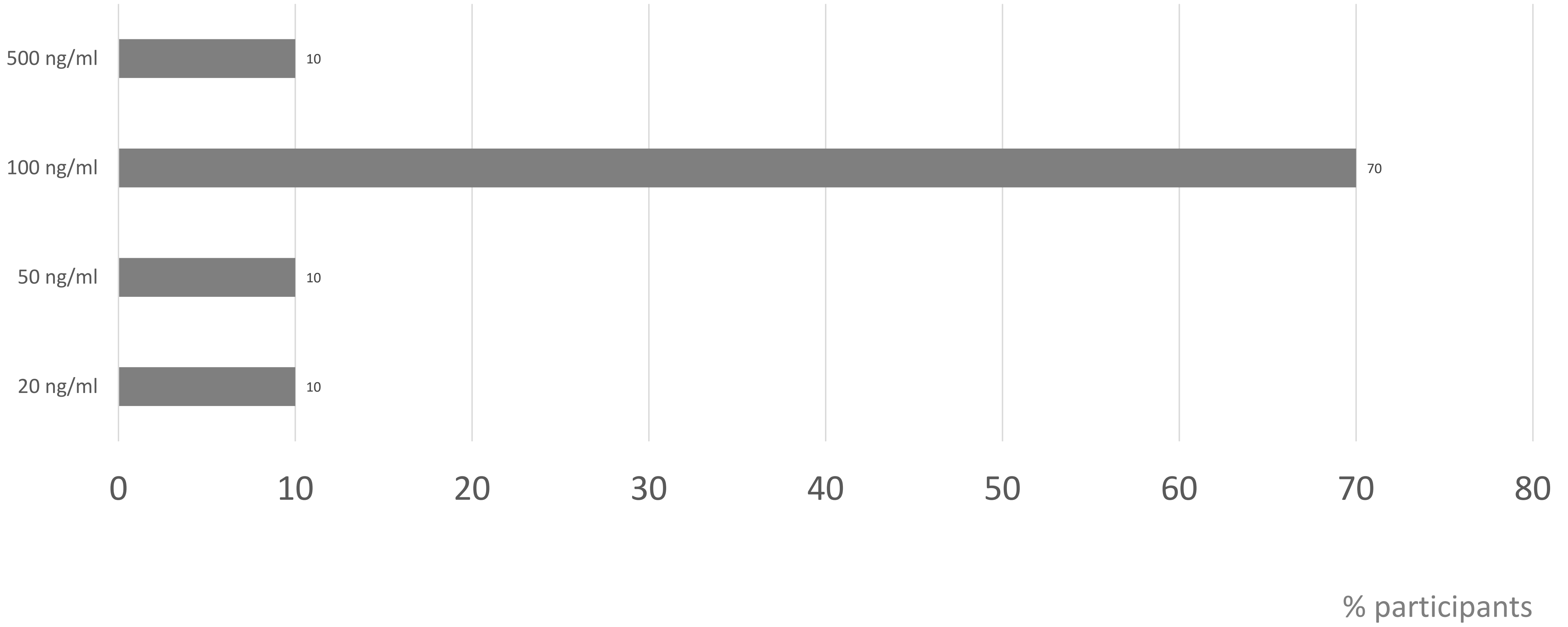

Question 5

If PSMA-PET/CT is used as imaging modality for staging (detection of metastases), the results must be systematically recorded using the PROMISE criteria.

**Agreement[1] (median)**	**Can't judge[2] (%)**
8.0	15

1 Score on a 9-point scale: 1-3 inappropriate (red), 4-6 uncertain or disagreement (yellow), 7-9 appropriate (green)
2 Total number of respondents=40

Question 6

How important are the following factors to determine whether imaging for staging (detection of metastases) is necessary?

Options	Importance[1] (median)	Can't judge[2] (%)
a. cT-stadium (clinical)	7.5	15
b. mT-stadium (MRI)	7.0	15
c. Gleason score	9.0	13
d. Presence of high % cribriform pattern in prostate biopsies	6.0	10
e. PSA value	8.0	5
f. PSA density	5.5	20
g. Vulnerable health	7.0	5
h. Symptoms	7.0	5

1 Score on a 9-point scale: 1-3 inappropriate (red), 4-6 uncertain or disagreement (yellow), 7-9 appropriate (green)
2 Total number of respondents=40

Consensus

Question 7a

Which imaging modality do you consider most suitable as additional examination in the following situations if osseous lesions are found during PSMA-PET/CT in a patient with high-risk prostate cancer? [1 answer option possible]
When in doubt whether these are metastases

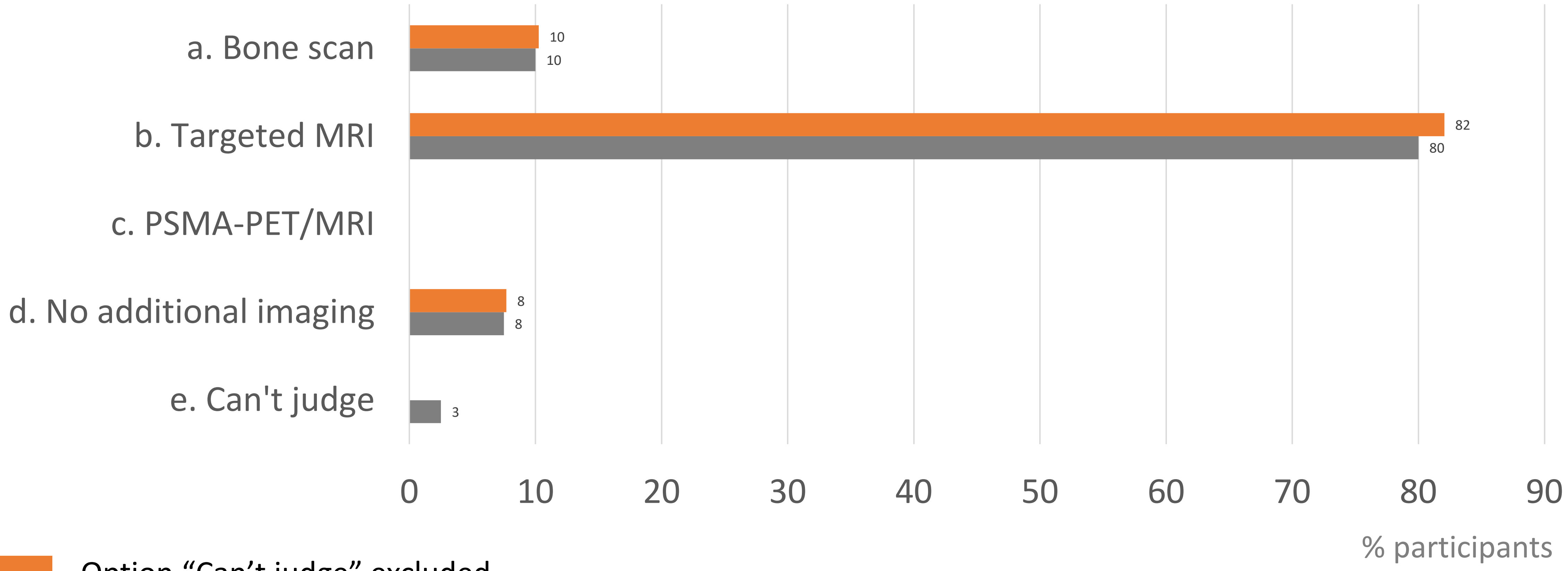

Option “Can’t judge” excluded

Question 7b

Which imaging modality do you consider most suitable as additional examination in the following situations if osseous lesions are found during PSMA-PET/CT in a patient with high-risk prostate cancer? [1 answer option possible]

If fracture is suspected

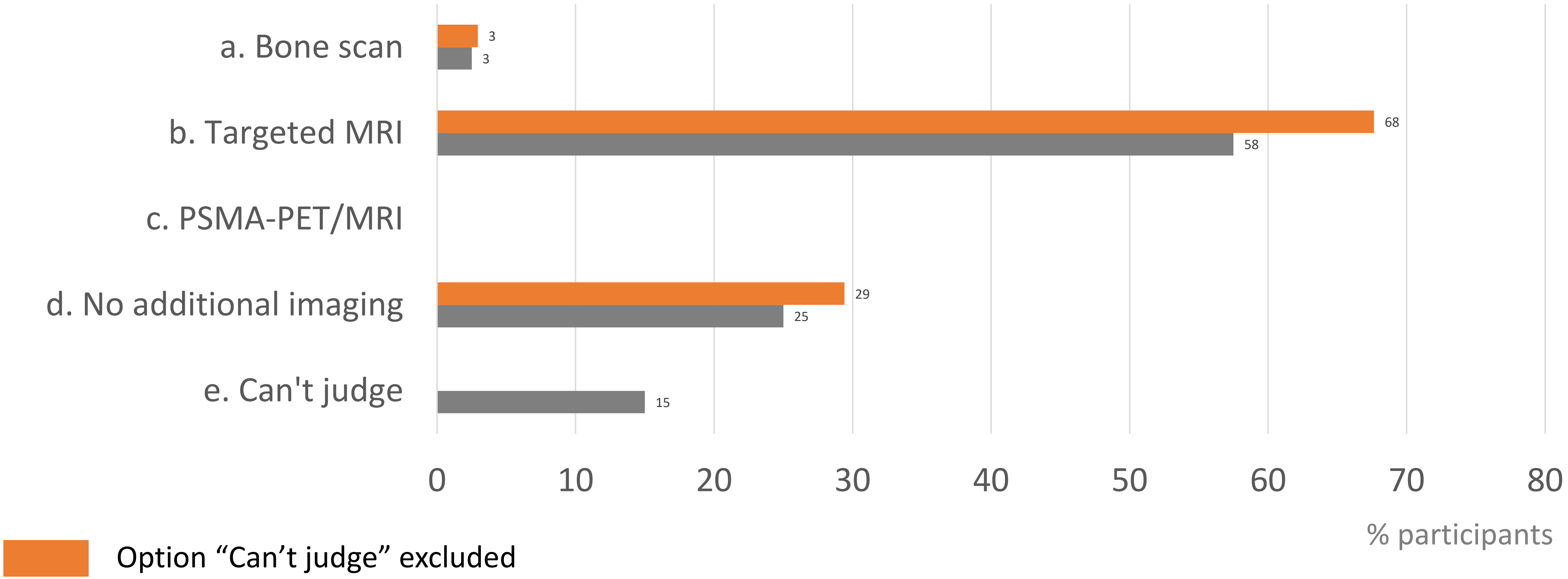

Consensus

Question 7c

Which imaging modality do you consider most suitable as additional examination in the following situations if osseous lesions are found during PSMA-PET/CT in a patient with high-risk prostate cancer? [1 answer option possible]

If spinal cord compression is suspected

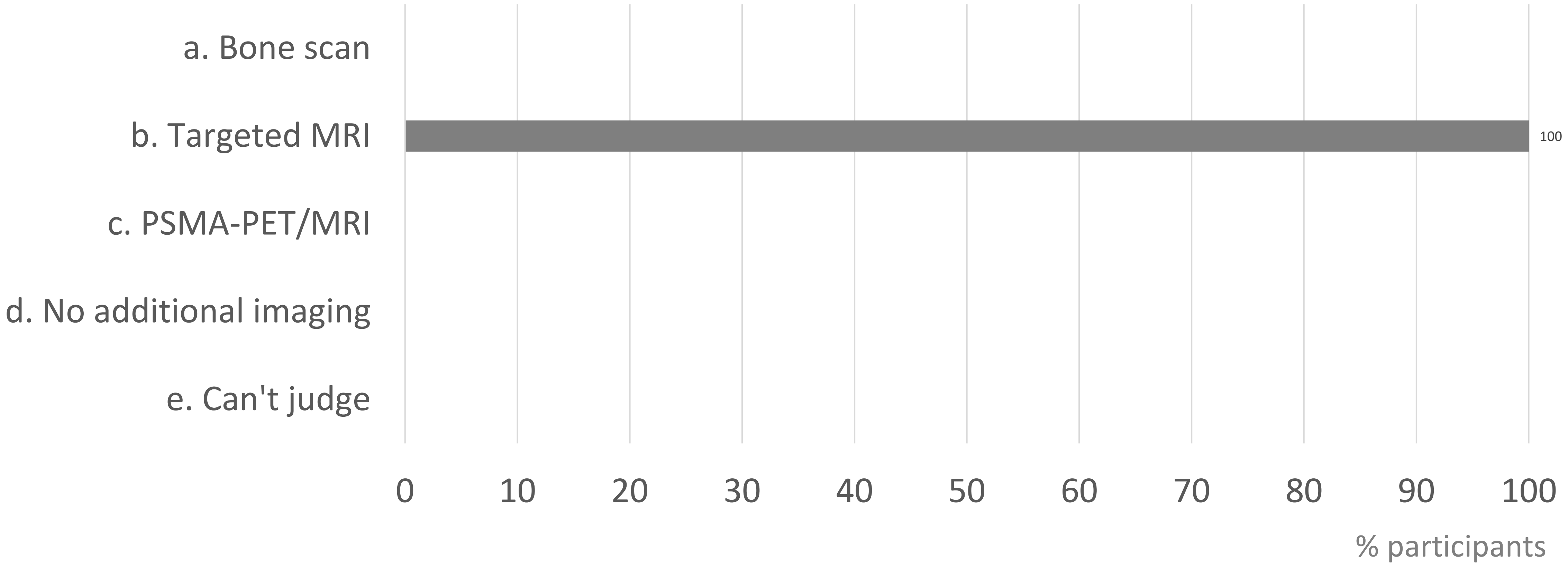

Part 2: interpretation diagnosis

Question 8 – Clinical case scenario

6 bone lesions are seen on a PSMA-PET/CT of a patient:

- 5 bone lesions are localised within the axial skeleton, of which 2 have an anatomical substrate on CT scan
- 1 bone lesion localised outside the axial skeleton with anatomical substrate on CT scan

How would you treat this patient? [1 answer option possible]

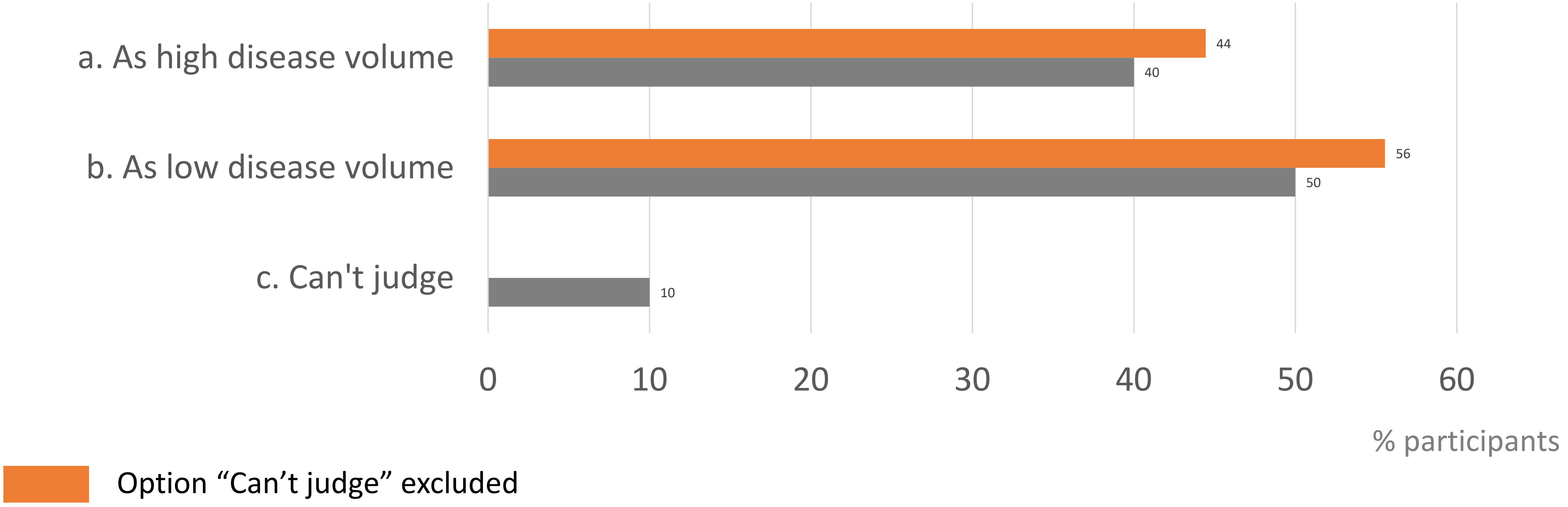

Question 9

Bone lesions on PSMA-PET without clear anatomical substrate on CT scan should not be considered as metastases.

Agreement[1] (median)	Can't judge[2] (%)
6.0	3

1 Score on a 9-point scale: 1-3 inappropriate (red), 4-6 uncertain or disagreement (yellow), 7-9 appropriate (green)
2 Total number of respondents=40

Question 10

If results are available from both conventional imaging and PSMA-PET/CT, and the results of the PSMA-PET/CT lead to ‘upstaging’, would you opt for a more intensified treatment*? [1 answer option possible]

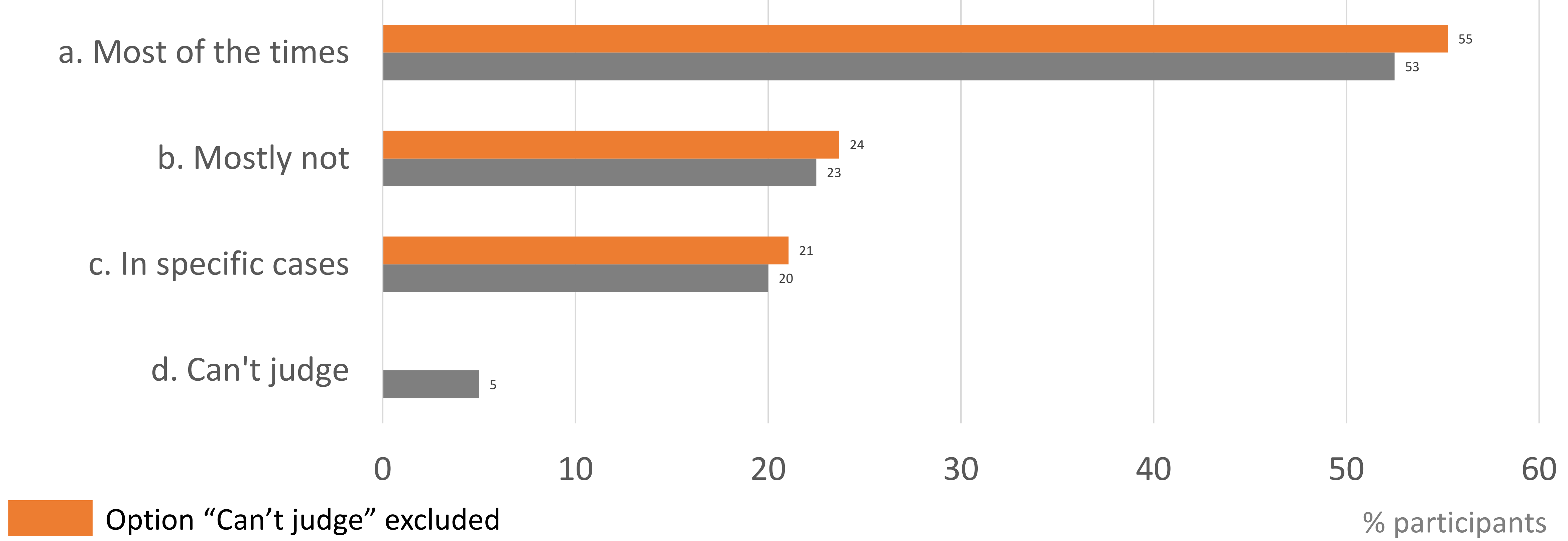

*e.g. ADT + ARPI + docetaxel instead of ADT + ARPI, or ADT + ARPI instead of ADT + prostate RT

Question 11

If results are available from both conventional imaging and PSMA-PET/CT, and the results of the PSMA-PET/CT lead to 'downstaging', would you opt for a less intensified treatment*? [1 answer option possible]

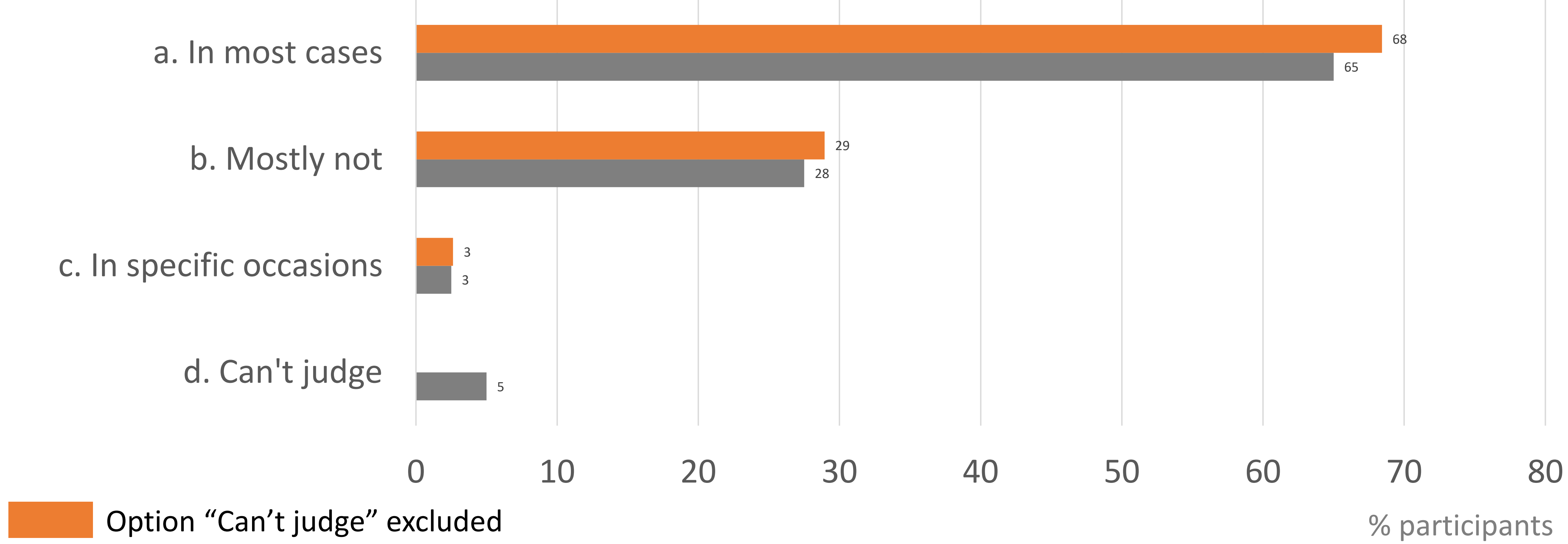

*e.g. ADT + ARPI instead of ADT + ARPI + docetaxel, or ADT + prostate RT instead of ADT + ARPI

Questions 12-13

Statements

	Agreement[1] (median)	Can't judge[2] (%)
PSMA-PET/CT is an appropriate option to determine treatment in patients with de novo mHSPC.	8.0	3
The CHAARTED definition for high-volume disease, based on conventional imaging, is also valid to interpret results of PSMA-PET/CT	6.0	3

1 Score on a 9-point scale: 1-3 inappropriate (red), 4-6 uncertain or disagreement (yellow), 7-9 appropriate (green)
2 Total number of respondents=40

Consensus

Question 14

There is a need in <u>clinical practice</u> for a new definition of disease extent based on PSMA-PET/CT. This definition can be based on different (combinations of) parameters. Which parameters should be part of this definition? [multiple options possible]

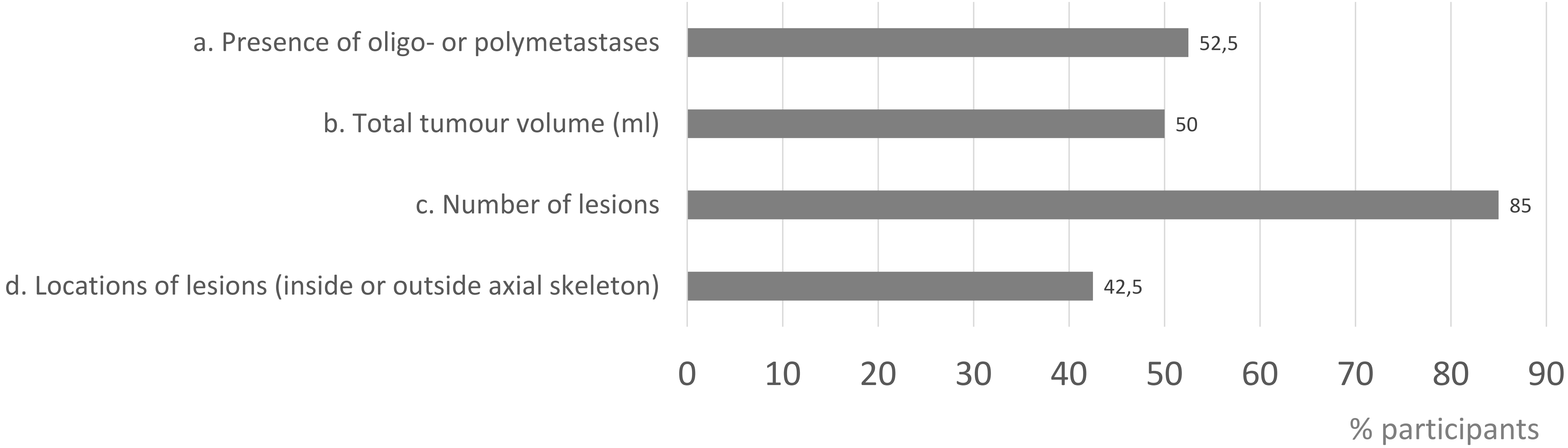

Question 14 – by specialty

There is a need in clinical practice for a new definition of disease extent based on PSMA-PET/CT. This definition can be based on different (combinations of) parameters. Which parameters should be part of this definition? [multiple options possible]

Parameters	Medical oncology (N=14) %	Urology (N=13) %	Radiology + Nuclear medicine (N=7) %	Radiotherapy (N=6) %
Oligo- or polymetastases	64	15	71	83
Total tumour volume	57	46	71	17
Number of lesions	71	100	86	83
Location of lesions	36	62	29	33

Question 14 – combinations

Oligo/poly metastases	Total tumour volume	Number of lesions	Location of lesions	Number of respondents	Total (%)
■				3	8
	■			-	-
		■		2	5
			■	-	-
■	■			3	8
■		■		3	8
■			■	-	-
	■	■		6	15
	■		■	-	-
		■	■	8	20
■	■	■		6	15
■	■		■	-	-
■		■	■	4	10
	■	■	■	3	8
■	■	■	■	2	5

Part 3: treatment choice

Clinical scenario 1

For all scenarios: all PSMA-avid lesions had anatomical substrate on CT.

Men, 70 years old

- No relevant medical history
- No use of comedication
- ECOG PS: 0
- PSA: 50 ng/ml
- DRE: T3 – mpMRI: T3a
- Prostate biopsy: Gleason score 4+4 (ISUP 4)
- PSMA-PET/CT:
  - Multiple pathologically enlarged lymph nodes (>10; max 2 cm short axis), also above iliac and aorta bifurcation
  - Clinical stage: cT3aN1M1a

Question 15

How would you classify this patient's disease?

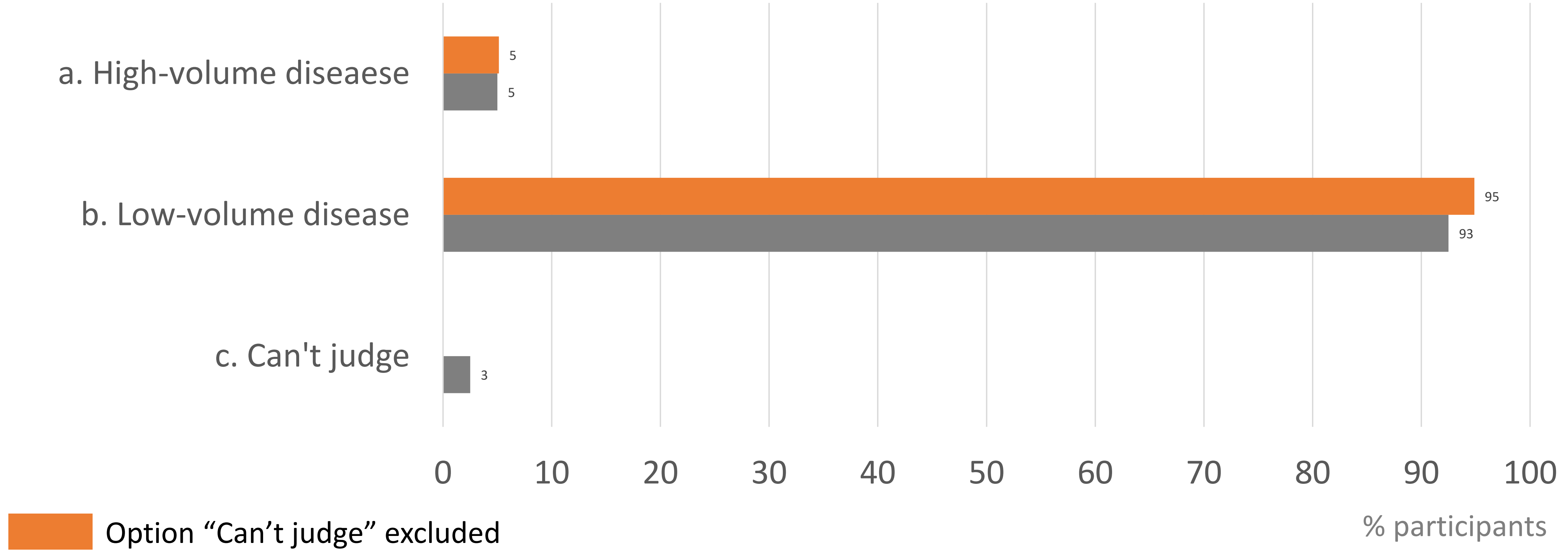

Question 16

How would you treat this patient?

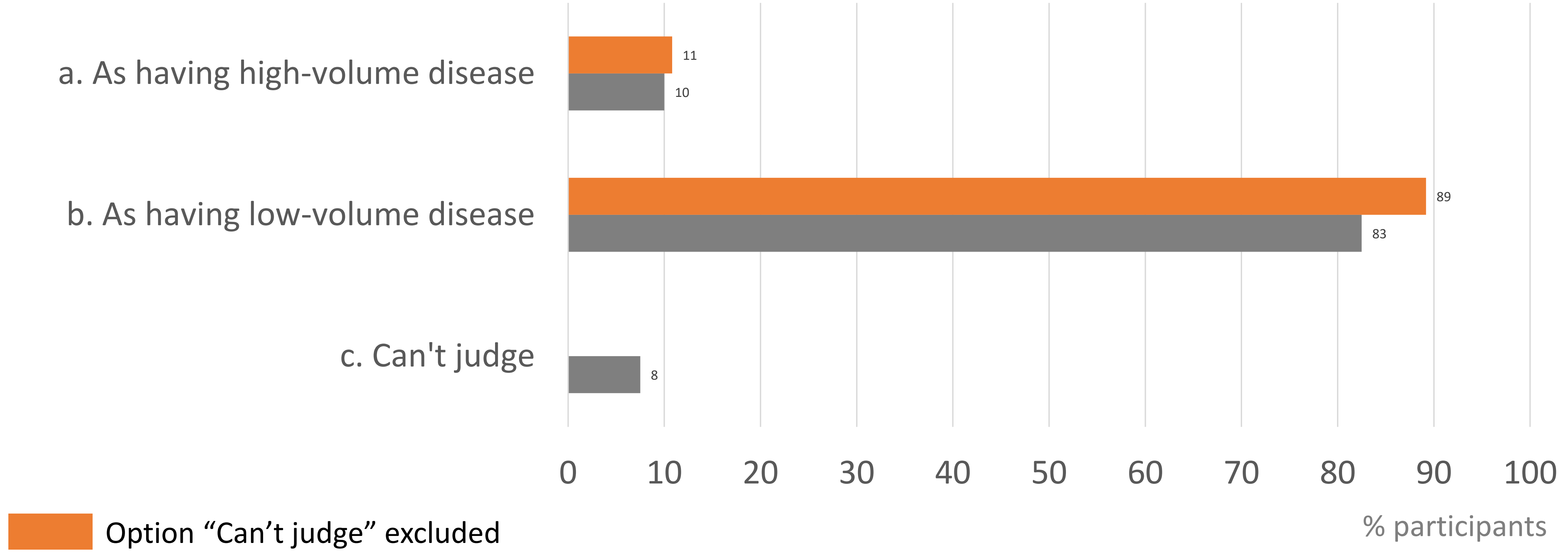

Question 17

How appropriate do you consider the following options for this patient?

	Appropriateness[1] (median)	Can't judge[2] (%)
a. ADT monotherapy	2.0	15
b. ADT + prostate RT	8.0	15
c. ADT + docetaxel	2.0	18
d. ADT + ARPI	8.0	18
e. ADT + ARPI + prostate RT	5.0	18
f. ADT + ARPI + docetaxel	1.0	18
g. ADT + prostate RT + ARPI + docetaxel	1.0	18

1 Score on a 9-point scale: 1-3 inappropriate (red), 4-6 uncertain or disagreement (yellow), 7-9 appropriate (green)
2 Total number of respondents=40

Clinical scenario 2

Men, 70 years old

- No relevant medical history
- No use of comedication
- ECOG PS: 0
- PSA: 50 ng/ml
- DRE: T3 – mpMRI: T3a
- Prostate biopsy: Gleason score 4+4 (ISUP 4)
- PSMA-PET/CT:
  - Multiple significantly enlarged pathologically bulky lymph nodes (short axis diameter 5-7 cm), also above the iliac and aorta bifurcation
- Clinical stage: cT3aN1M1a

Consensus

Question 18

How would you classify this patient’s disease?

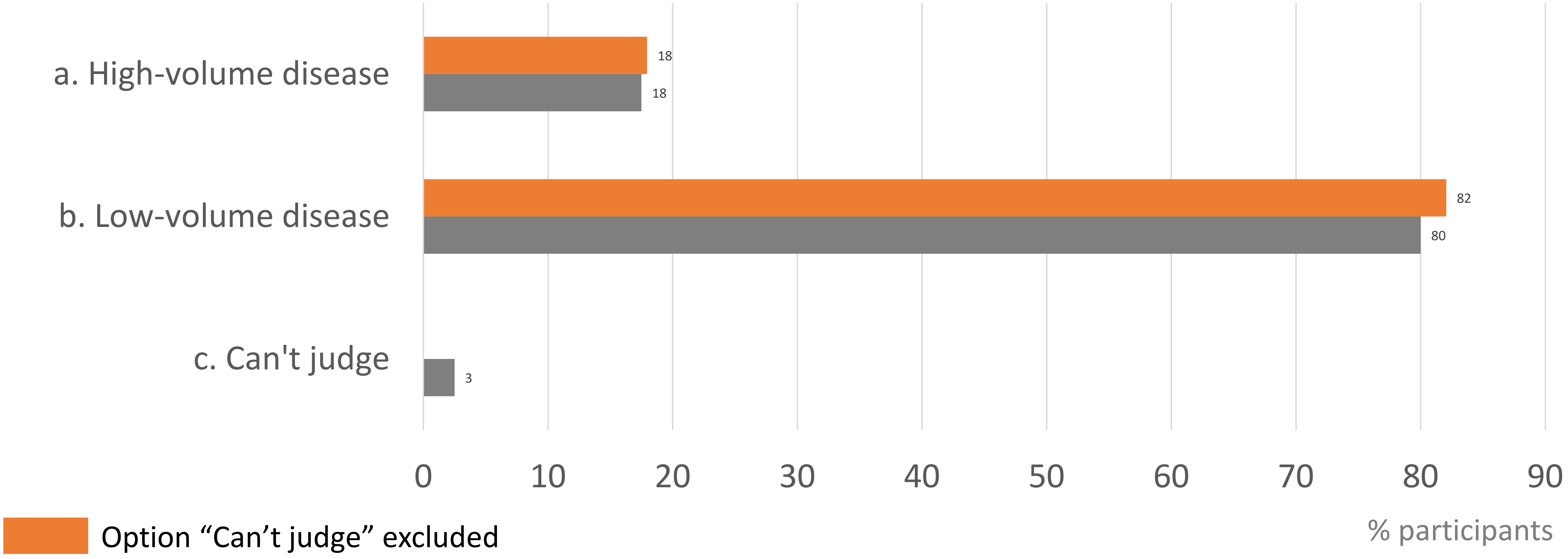

Question 19

How would you treat this patient?

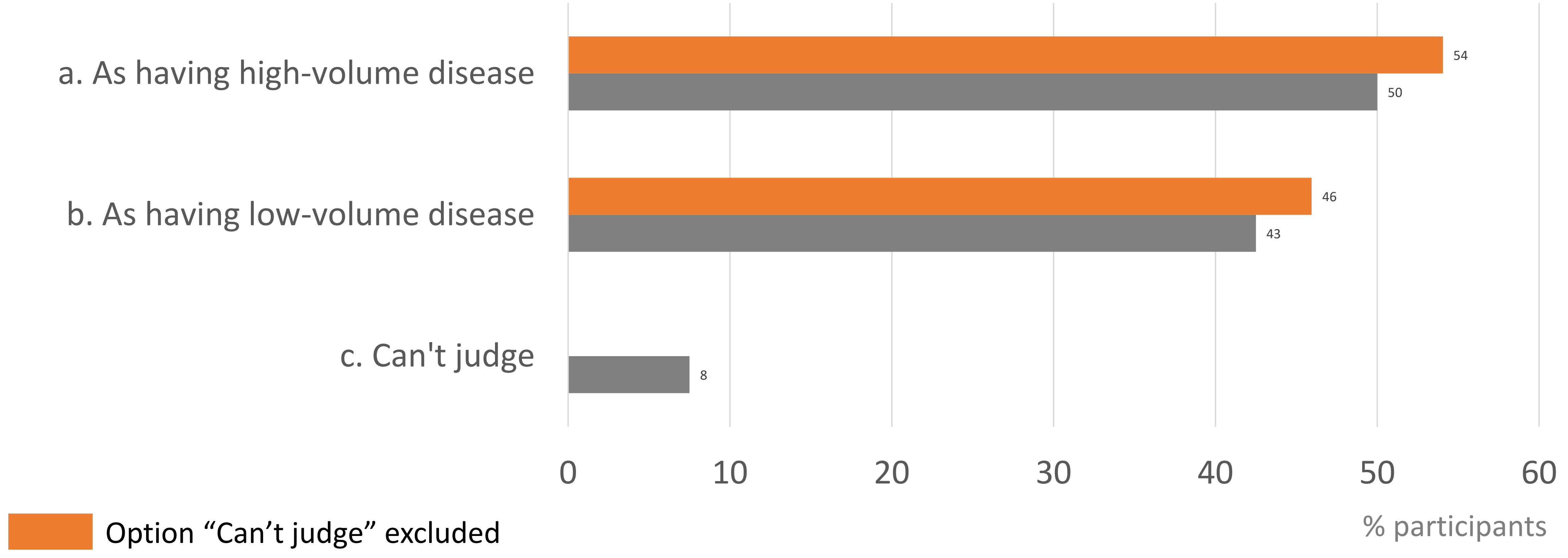

Question 20

How appropriate do you consider the following options for this patient?

	Appropriateness[1] (median)	Can't judge[2] (%)
a. ADT monotherapy	1.0	15
b. ADT + prostate RT	6.0*	18
c. ADT + docetaxel	4.0	18
d. ADT + ARPI	8.0	18
e. ADT + ARPI + prostate RT	5.0	18
f. ADT + ARPI + docetaxel	3.0	18
g. ADT + prostate RT + ARPI + docetaxel	1.0	18

1 Score on a 9-point scale: 1-3 inappropriate (red), 4-6 uncertain or disagreement (yellow), 7-9 appropriate (green)
2 Total number of respondents=40
*Disagreement: at least one-third of the scores in each of the sections 1-3 and 7-9

Clinical scenario 3

Men, 70 years old

- No relevant medical history
- No use of comedication
- ECOG PS: 0
- PSA: 50 ng/ml
- DRE: T3 – mpMRI: T3a
- Prostate biopsy: Gleason score 4+4 (ISUP 4)
- PSMA-PET/CT:
  - 2 bone metastases (L1, acetabulum)
  - 2 locoregional lymph nodes, para-iliacal, below the iliac bifurcation
- Clinical stage: cT3aN1M1b

Consensus

Question 21

How would you classify this patient's disease?

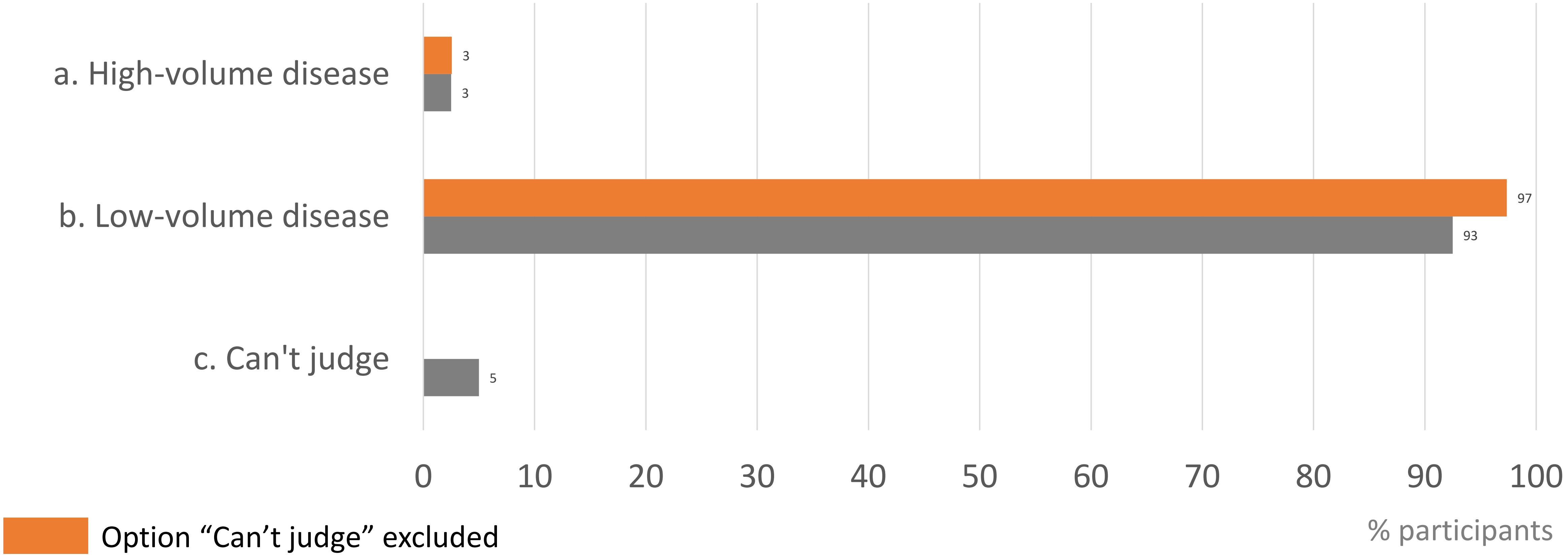

Consensus

Question 22

How would you treat this patient?

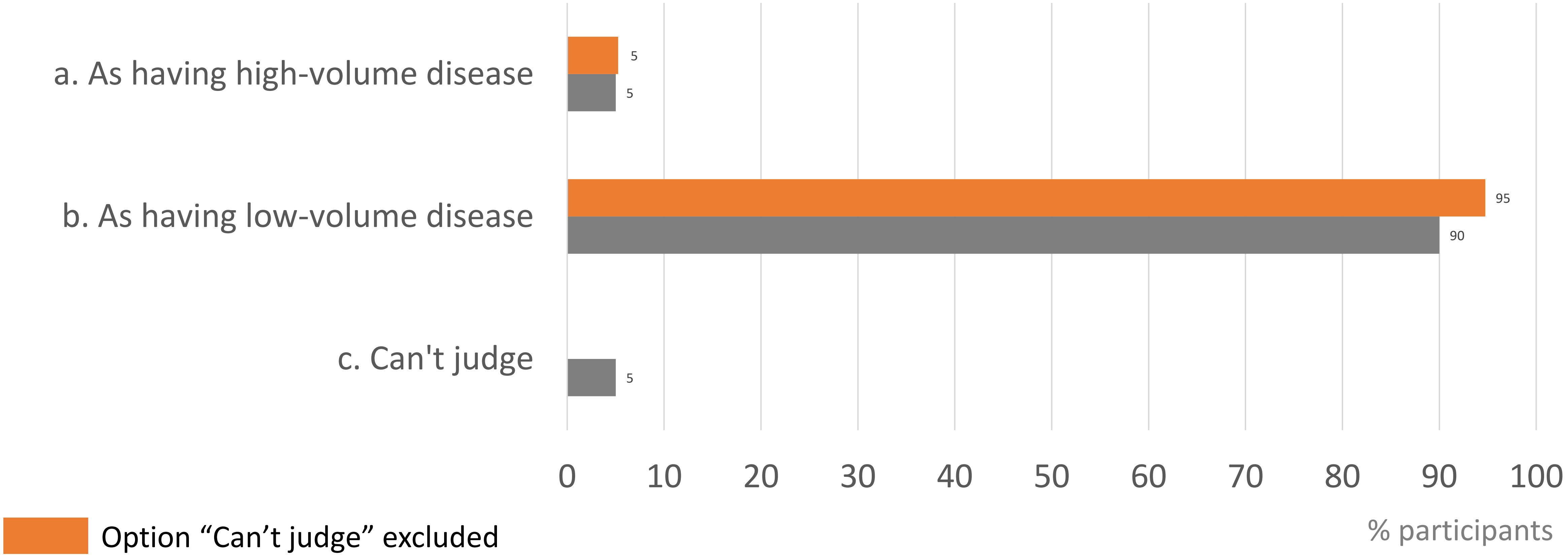

Question 23

How appropriate do you consider the following options for this patient?

	Appropriateness[1] (median)	Can't judge[2] (%)
a. ADT monotherapy	1.0	18
b. ADT + prostate RT	8.0	18
c. ADT + docetaxel	2.0	18
d. ADT + ARPI	8.0	18
e. ADT + ARPI + prostate RT	6.0*	18
f. ADT + ARPI + docetaxel	2.0	18
g. ADT + prostate RT + ARPI + docetaxel	1.0	18
h. Metastasis-directed therapy ± prostate RT ± systemic therapy	2.0	18

1 Score on a 9-point scale: 1-3 inappropriate (red), 4-6 uncertain or disagreement (yellow), 7-9 appropriate (green)

2 Total number of respondents=40

*Disagreement: at least one-third of the scores in each of the sections 1-3 and 7-9

Clinical scenario 4

Men, 70 years old

- No relevant medical history
- No use of comedication
- ECOG PS: 0
- PSA: 50 ng/ml
- DRE: T3 – mpMRI: T3a
- Prostate biopsy: Gleason score 4+4 (ISUP 4)
- PSMA-PET/CT:
  - 2 bone metastases (L1, acetabulum)
  - 2 locoregional lymph nodes, para-iliacal, below iliac bifurcation
  - 2 lung metastases
- Clinical stage: cT3aN1M1c

Question 24

How would you classify this patient's disease?

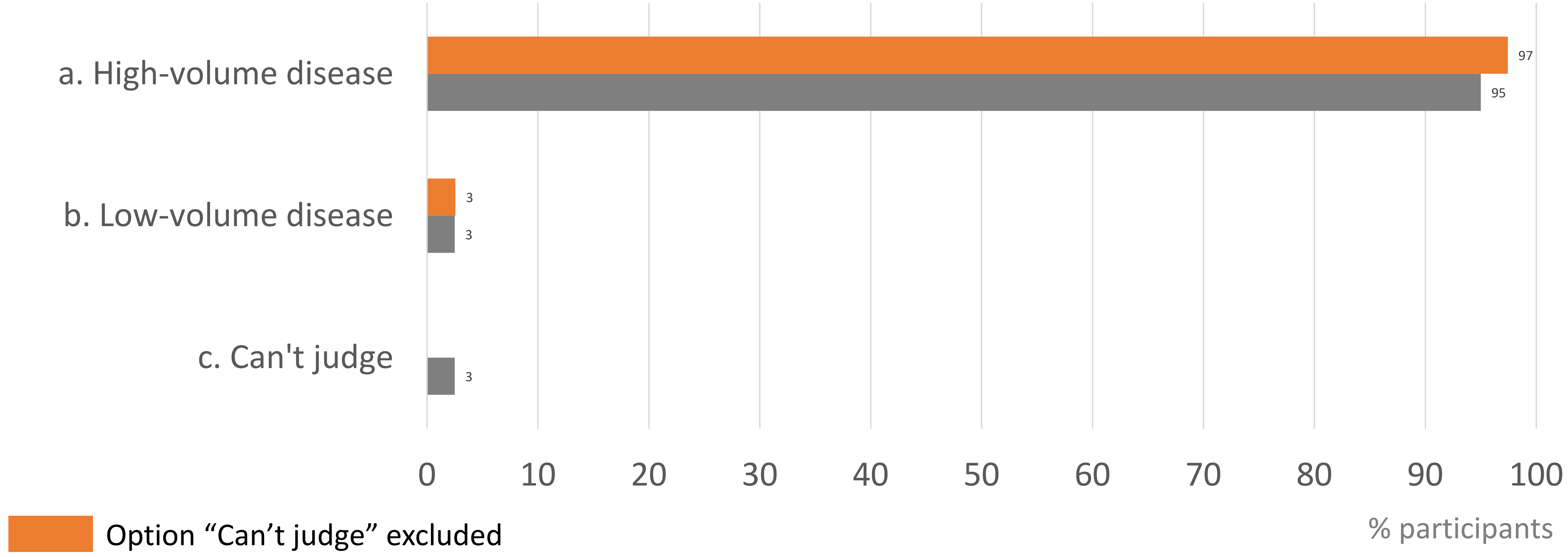

Consensus

Question 25

How would you treat this patient?

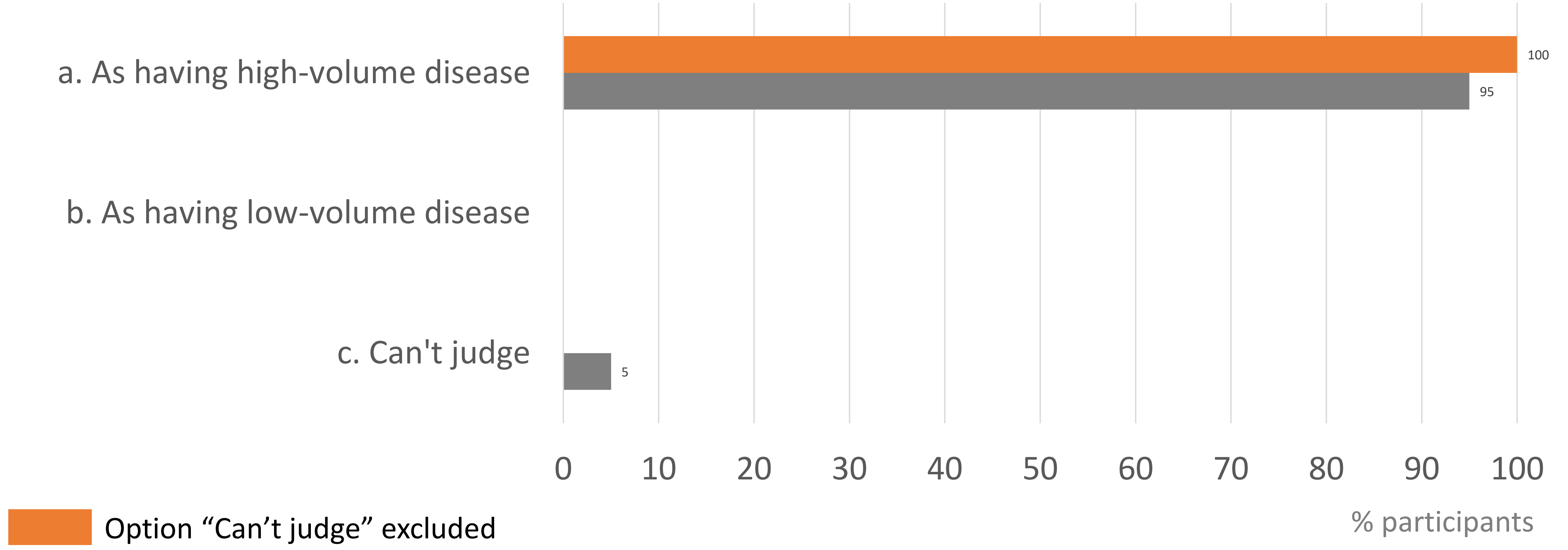

Question 26

How appropriate do you consider the following options for this patient?

	Appropriateness[1] (median)	Can't judge[2] (%)
a. ADT monotherapy	1.0	15
b. ADT + prostate RT	1.0	15
c. ADT + docetaxel	7.0	15
d. ADT + ARPI	8.0	15
e. ADT + ARPI + prostate RT	1.5	15
f. ADT + ARPI + docetaxel	8.0	18
g. ADT + prostate RT + ARPI + docetaxel	1.0	18
h. Metastasis-directed therapy ± prostate RT ± systemic therapy	1.0	18

1 Score on a 9-point scale: 1-3 inappropriate (red), 4-6 uncertain or disagreement (yellow), 7-9 appropriate (green)
2 Total number of respondents=40

Clinical scenario 5

Men, 70 years old

- No relevant medical history
- No use of comedication
- ECOG PS: 0
- PSA: 50 ng/ml
- DRE: T3 – mpMRI: T3a
- Prostate biopsy: Gleason score 4+4 (ISUP 4)
- PSMA-PET/CT:
  - 2 bone metastases (L1, acetabulum)
  - 2 locoregional lymph nodes, para-iliacal, below iliac bifurcation
  - 2 liver metastases
- Clinical stage: cT3aN1M1c

Question 27

How would you classify this patient's disease?

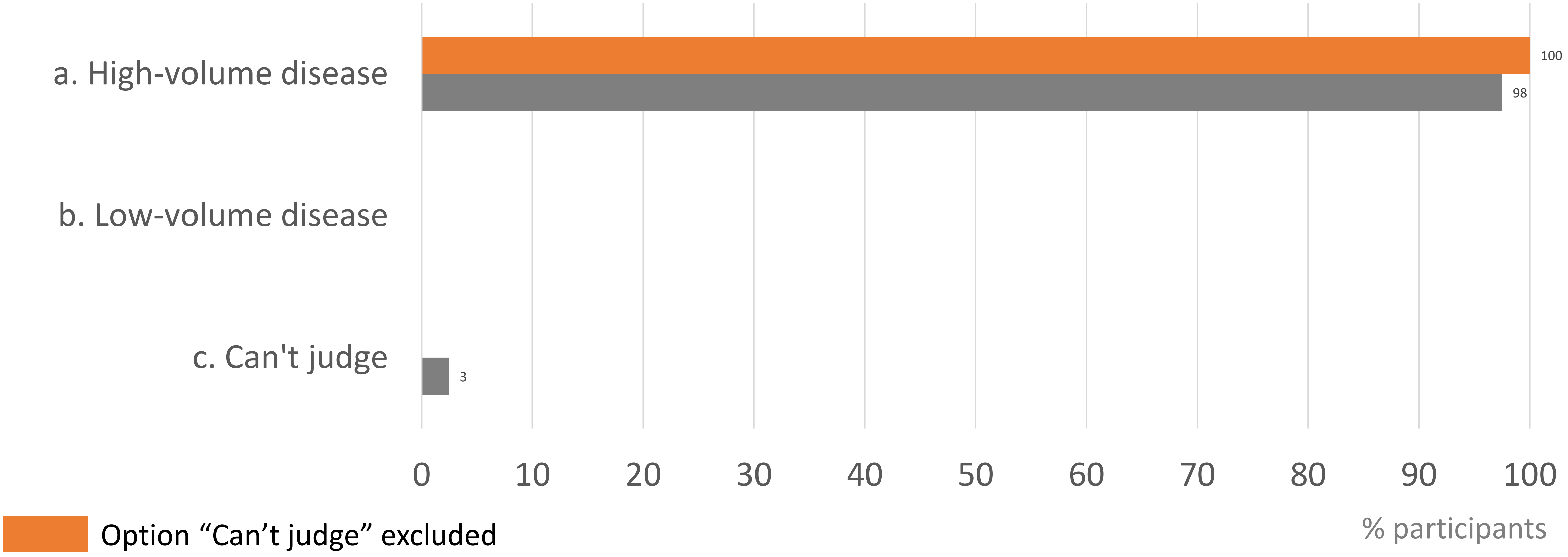

Question 28

How would you treat this patient?

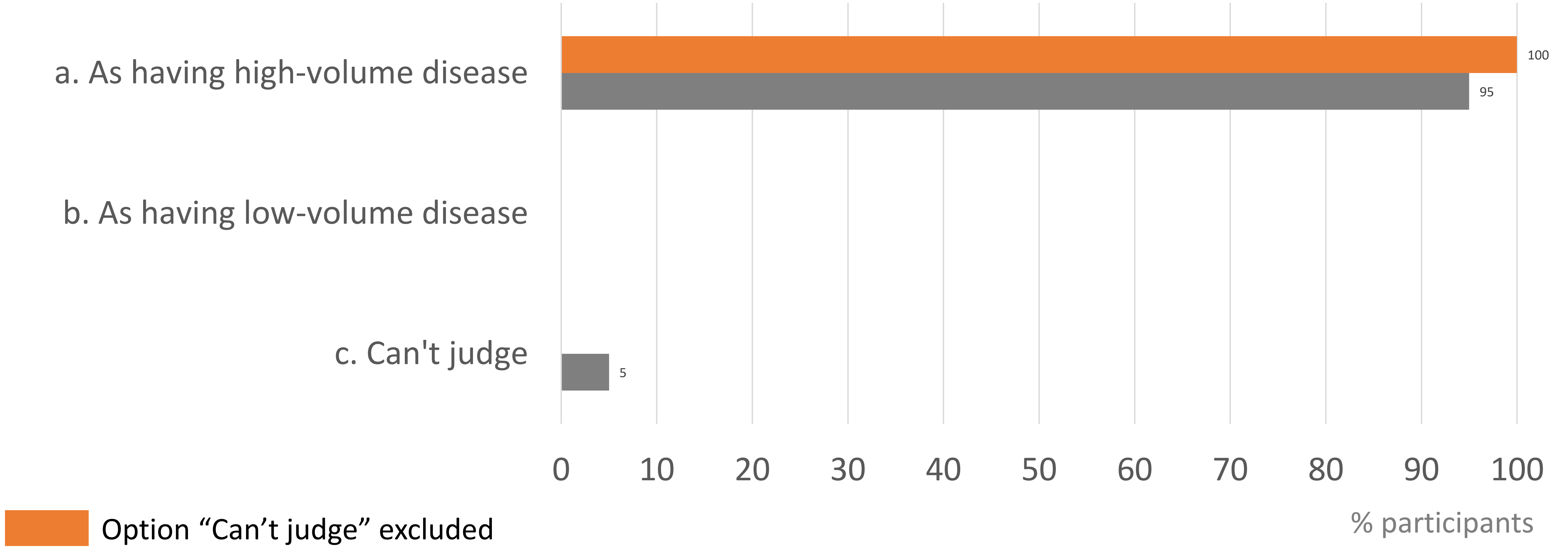

Question 29

How appropriate do you consider the following options for this patient?

	Appropriateness[1] (median)	Can't judge[2] (%)
a. ADT monotherapy	1.0	15
b. ADT + prostate RT	1.0	15
c. ADT + docetaxel	7.0	18
d. ADT + ARPI	7.0	18
e. ADT + ARPI + prostate RT	1.0	18
f. ADT + ARPI + docetaxel	9.0	18
g. ADT + prostate RT + ARPI + docetaxel	1.0	18

1 Score on a 9-point scale: 1-3 inappropriate (red), 4-6 uncertain or disagreement (yellow), 7-9 appropriate (green)
2 Total number of respondents=40

Clinical scenario 6

Men, 70 years old

- No relevant medical history
- No use of comedication
- ECOG PS: 0
- PSA: 50 ng/ml
- DRE: T3 – mpMRI: T3a
- Prostate biopsy: Gleason score 4+4 (ISUP 4)
- PSMA-PET/CT:
  - 6 bone metastases (5 vertebral metastases L1-4, S1 and 1 metastasis in the left proximal humerus)
  - Multiple locoregional lymph nodes, all below iliac bifurcation
  - Clincial stage: cT3aN1M1b

Question 30

Consensus

How would you classify this patient's disease?

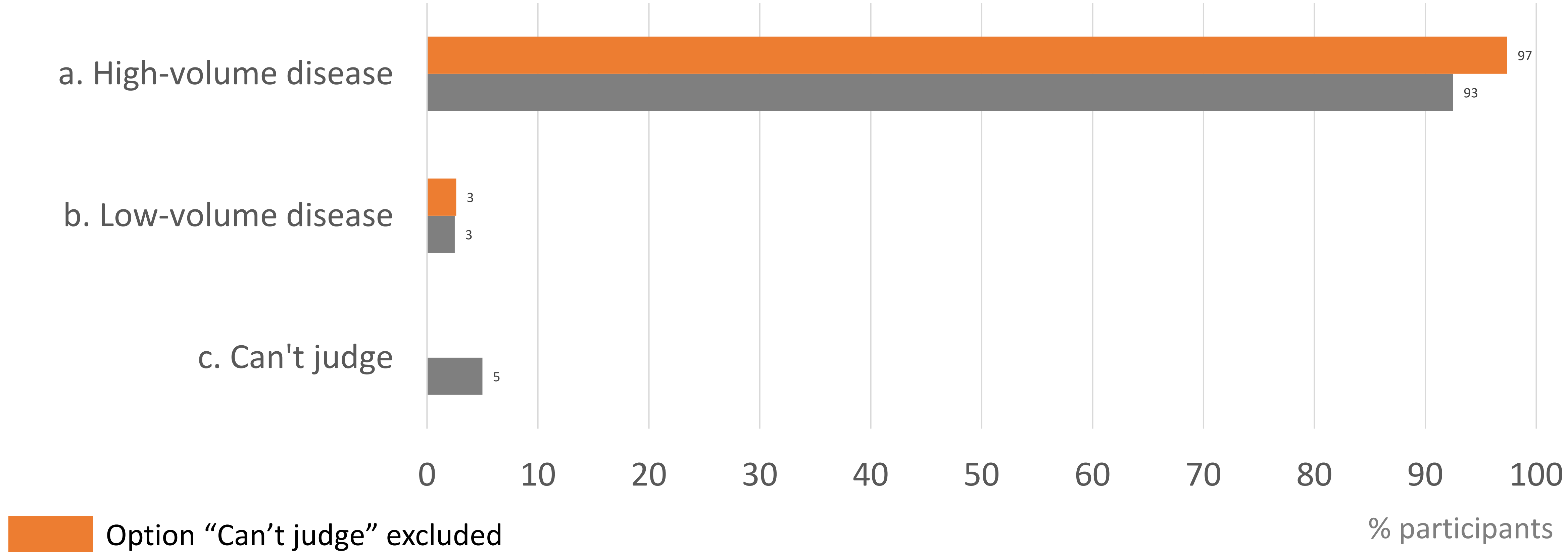

Consensus

Question 31

How would you treat this patient?

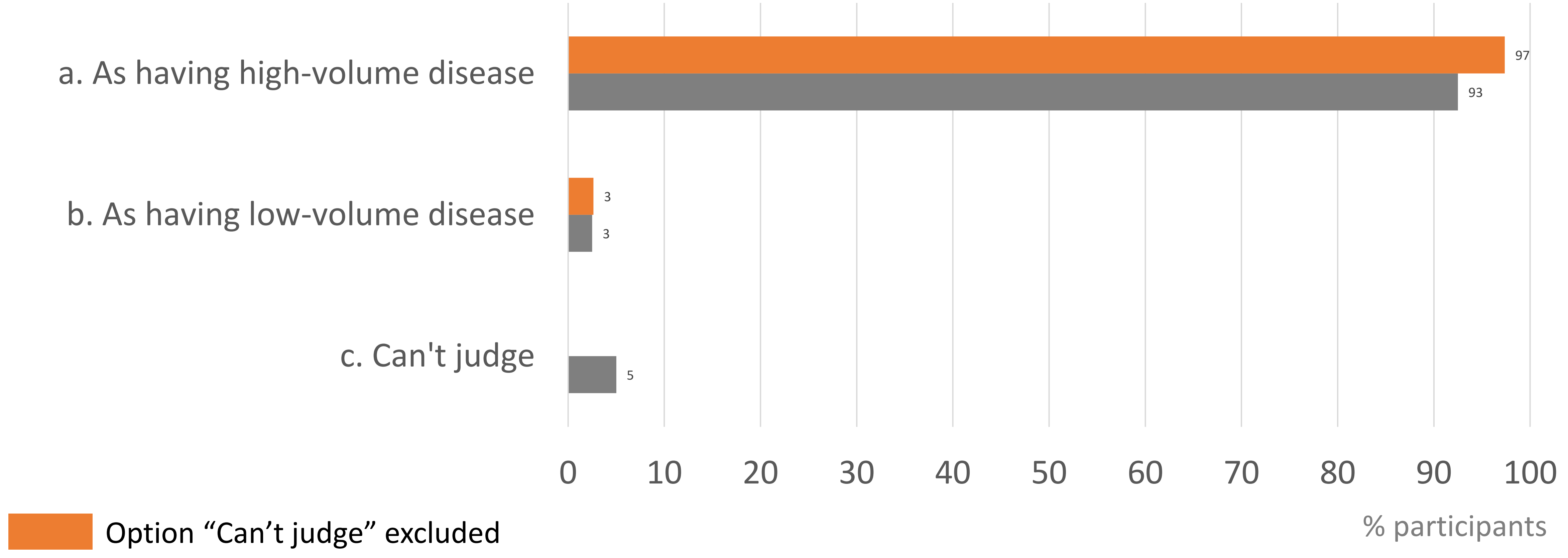

Question 32

How appropriate do you consider the following options for this patient?

	Appropriateness[1] (median)	Can't judge[2] (%)
a. ADT monotherapy	1.0	15
b. ADT + prostate RT	1.0	15
c. ADT + docetaxel	7.0	18
d. ADT + ARPI	8.0	18
e. ADT + ARPI + prostate RT	2.0	18
f. ADT + ARPI + docetaxel	8.0	18
g. ADT + prostate RT + ARPI + docetaxel	2.0	18

1 Score on a 9-point scale: 1-3 inappropriate (red), 4-6 uncertain or disagreement (yellow), 7-9 appropriate (green)
2 Total number of respondents=40

Clinical scenario 7

Men, 70 years old

- No relevant medical history
- No use of comedication
- ECOG PS: 0
- PSA: 50 ng/ml
- DRE: T3 – mpMRI: T3a
- Prostate biopsy: Gleason score 4+4 (ISUP 4)
- PSMA-PET/CT:
  - 10 bone metastases (all within axial skeleton)
  - Multiple locoregional lymph nodes, all below iliac bifurcation
  - Clinical stage: cT3aN1M1b

Question 33

How would you classify this patient's disease?

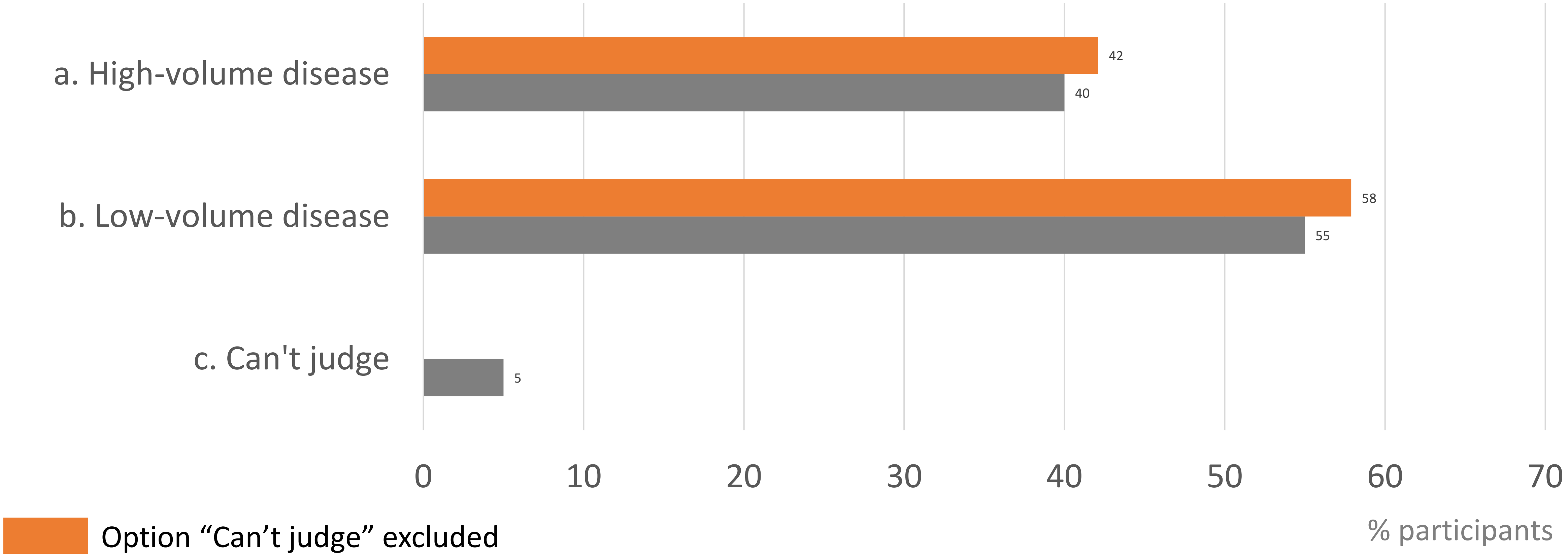

Consensus

Question 34

How would you treat this patient?

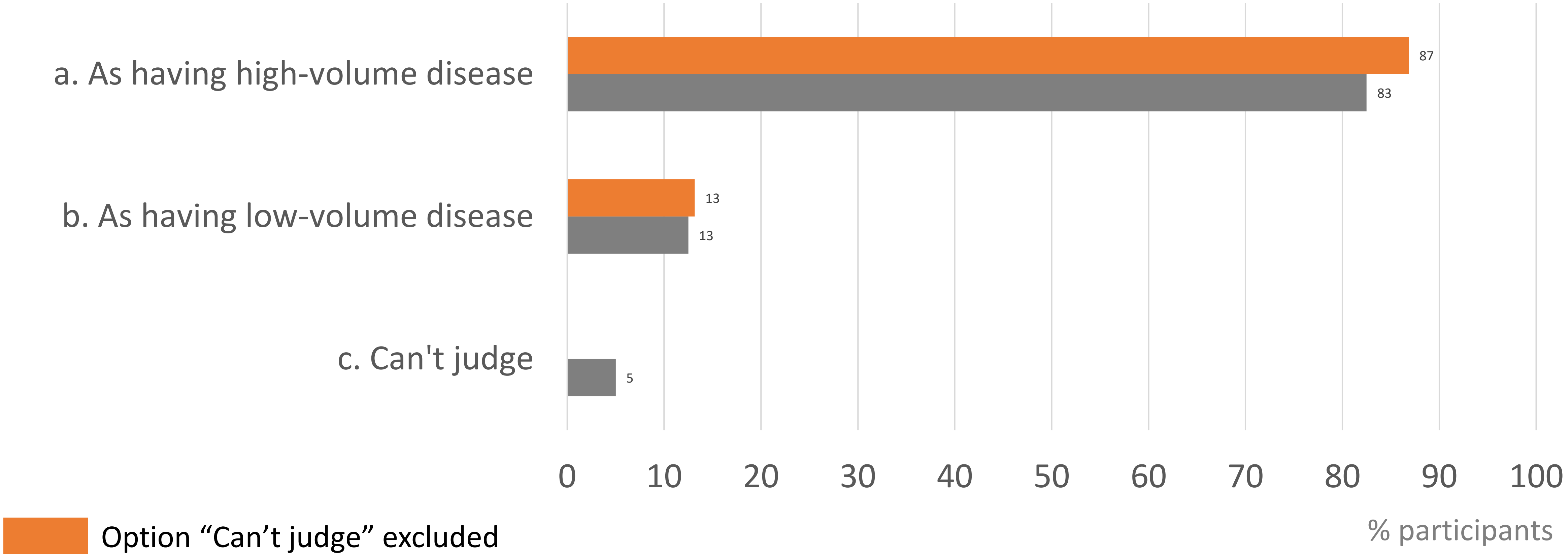

Question 35

How appropriate do you consider the following options for this patient?

	Appropriateness[1] (median)	Can't judge[2] (%)
a. ADT monotherapy	1.0	15
b. ADT + prostate RT	2.0	15
c. ADT + docetaxel	7.0	18
d. ADT + ARPI	8.0	18
e. ADT + ARPI + prostate RT	2.0	18
f. ADT + ARPI + docetaxel	8.0	18
g. ADT + prostate RT + ARPI + docetaxel	2.0	18

1 Score on a 9-point scale: 1-3 inappropriate (red), 4-6 uncertain or disagreement (yellow), 7-9 appropriate (green)
2 Total number of respondents=40

Question 36

How important do you consider the following parameters (other than disease characteristics like PSA value and Gleason score) to choose between ARPIs

Options	Importance[1] (median)	Can't judge[2] (%)
a. Presence of comorbidities	8.0	23
b. Use of comedication	8.0	25
c. ECOG PS	7.0	23
d. Patient age	3.0	23
e. Life expectancy	7.0	20
f. Frailty (elderly)	7.0	20
g. Pain/discomfort of local disase	3.0	23
h. Pain/discomfort of metastases	3.0	23

1 Score on a 9-point scale: 1-3 inappropriate (red), 4-6 uncertain or disagreement (yellow), 7-9 appropriate (green)

2 Total number of respondents=40

Question 37

The different ARPIs* are equivalent for the treatment of:

Options	Appropriateness[1] (median)	Can't judge[2] (%)
a. Low-volume disease (CHAARTED)	7.0	35
b. High-volume disease (CHAARTED)	7.5	35
c. Low-risk disease (LATITUDE)	7.0	38
d. High-risk disease (LATITUDE)	7.5	40

1 Score on a 9-point scale: 1-3 inappropriate (red), 4-6 uncertain or disagreement (yellow), 7-9 appropriate (green)
2 Total number of respondents=40

*abiraterone, apalutamide, enzalutamide if ADT + ARPI or abiraterone, darolutamide if ADT + ARPI + docetaxel. The choice of the ARPI should be in accordance with the applicable registration.
